# Trivial-nontrivial programmable topological metasurfaces for sensing and communication

**DOI:** 10.1038/s41377-026-02419-x

**Published:** 2026-08-20

**Authors:** Qiang Xiao, Long Chen, Qian Ma, Yu Ming Ning, Zi Xuan Cai, Yi Zhang, Ze Gu, Jian Wei You, Din Ping Tsai, Tie Jun Cui

**Affiliations:** 1 State Key Laboratory of Millimeter Waves, Southeast University, Nanjing, 210096 China; 2 Institute of Electromagnetic Space, Southeast University, Nanjing, 210096 China; 3 Department of Electrical Engineering and State Key Laboratory of Optical Quantum Materials, City University of Hong Kong, Kowloon, 999077 Hong Kong SAR China; 4 State Key Laboratory of Terahertz and Millimeter Waves, City University of Hong Kong, Hong Kong, 999077 China; 5 Department of Physics, City University of Hong Kong, Kowloon, 999077 Hong Kong SAR China

**Keywords:** Metamaterials, Photonic devices

## Abstract

Trivial-nontrivial topological switching provides a distinctive physical pathway for multifunctional electromagnetic systems, yet has never been exploited for integrated sensing and communication (ISAC). Existing ISAC architectures rely almost exclusively on trivial spatial-wave beamforming, constraining near-field sensing robustness and limiting hardware scalability in 6 G scenarios. Here, we propose an intelligent ISAC platform enabled by a programmable topological metasurface (PTM) that dynamically switches between trivial radiation states and non-trivial valley-Hall states, which is achieved through FPGA-controlled symmetry modulation of the PTM’s unit cells. In its non-trivial state, the PTM forms multiple topologically protected domain-wall channels, guiding surface waves with robustness and enabling the extraction of electromagnetic signatures for human localization. A convolutional neural network trained on these signatures achieves a localization accuracy of 99.54%. Upon position recognition, the PTM transitions to its trivial radiation phase, generating spatial phase-gradient beams for directional wireless communication without requiring additional hardware. Experimental results are consistent with theoretical predictions, validating an implementation of topological state switching for dual-mode ISAC functionality. It suggests that topological state programmability could be a potential mechanism for building compact, robust, and intelligent electromagnetic platforms for next-generation wireless systems.

## Introduction

Driven by the rapid evolution of wireless communication networks and advanced sensor technologies, there is an increasing demand for multifunctional platforms capable of concurrent data transmission and environmental sensing^1–3^. Integrated sensing and communication (ISAC) technology is a key candidate of the sixth-generation (6 G) wireless communications, promising unified spectrum and hardware reuse for simultaneous electromagnetic (EM) sensing and communication, and supporting advanced applications such as intelligent environment reconfiguration, and enhanced network performance^4–7^. The advent of programmable metasurfaces—ultrathin arrays of digitally tunable meta-atoms—has introduced unprecedented flexibility in manipulating the EM wavefronts for both sensing and communication tasks for dynamic propagation environment shaping, significantly boosting channel estimation and sensing accuracy in complex scenarios^8–17^. However, most existing ISAC platforms have been focused exclusively on spatial-wave beamforming, overlooking the rich potential of surface-wave regulation in near-field sensing applications.

Topological insulators (TIs) have garnered significant attention in recent years due to their unique and non-trivial band topology and potential applications of topological edge states^18^. Since the discovery of quantum Hall effect^19^, the intricate topological phase diagrams have unfolded like a lush tree, revealing a wealth of fascinating physical phenomena^20–23^. The confluence of topology and photonics has led to significant breakthroughs^24–27^, with photonic systems offering numerous advantages for topological physics and technical applications. Consequently, topological photonics has emerged as an extension of topological materials in optically engineered structures, bringing about revolutionary changes in optical science and technology. For instance, various intriguing topological phenomena such as quantum Hall TIs^28–30^, quantum spin Hall TIs^31–35^, and Floquet TIs^36–38^ have been observed in a range of photonic systems. Among them, insulating materials exhibiting topological valley-Hall insulating phases have attracted considerable interests^39–46^, demonstrating numerous intriguing phenomena related to valley symmetry, including the photonic valley-Hall effect, valley-locked transport, and valley-polarized states. However, the existing studies primarily achieve specific static topological EM properties with very limited reconfigurability, confining the guiding pathways for wave propagation to the edges and failing to fully utilize the vast internal space of TIs, thereby restricting the development of integrated applications. Recent research has reported the realization of highly integrated electrically reconfigurable quantum valley Hall insulators driven by a field-programmable gate array (FPGA)^47–50^, providing feasibility for complex real-time applications. While topological metasurfaces offer robust surface-wave control, the dynamic switch between nontrivial topological states for sensing and trivial radiative states for communication remains unexplored. Here, “nontrivial” denotes a valley-Hall topological state that supports protected domain-wall transport of surface waves, while “trivial” denotes the conventional radiative state without the topological protection, where the wave manipulation is achieved by phase-coded beamforming.

In this study, we present an adaptive ISAC platform leveraging a programmable topological metasurface (PTM) for human positioning and wireless communication. The PTM dynamically switches between a nontrivial topological state, constructing robust surface-wave channels for deep-learning-driven human positioning, and a trivial radiative state, enabling far-field beamforming for directional wireless communication. Specifically, in the nontrivial state, we engineer surface wave propagation to construct distinct domain-wall interfaces, each with a Vivaldi antenna for detecting human positions. The localized fields from these interfaces interact with human bodies, generating unique spectral responses analyzed by a convolutional neural network (CNN) trained on 111,000 spectral data points, achieving the human location accuracy of 99.54%. More broadly, recent advances in machine learning and material informatics have demonstrated the growing power of data-driven methods in metamaterials and photonic systems, including inverse design of thermal metamaterials, radiative-cooling meta-coatings, emissivity engineering, nonreciprocal thermal radiation, and optimized resonant emitters^51–55^. Inspired by these developments, here we employ CNN as an efficient data-driven tool to extract the discriminative spectral features and enable real-time human-position recognition in the proposed PTM platform. Upon identifying the human position, the PTM transitions to the trivial radiative state, directing spatial waves to establish wireless communication links. This unified approach eliminates the need for separate hardware, simplifying the system architecture, reducing costs, and facilitating scalable deployment in 6 G, smart cities, healthcare, and intelligent wireless networks. A central challenge in this unified architecture is that the sensing and communication functions rely on fundamentally different electromagnetic mechanisms: the former requires the transmission of topologically protected surface-wave transport along programmable domain-wall routes, whereas the latter requires the radiative phase-gradient control of spatial waves. It is nontrivial to integrate these two mechanisms into the same metasurface hardware and avoid the mutual interference, so as to maintain a closed-loop sensing-recognition-communication workflow. In this work, we address this challenge through FPGA-controlled trivial-nontrivial state switching, which enables the PTM to execute the two functions sequentially on demand rather than simultaneously in a fixed physical state.

## Results

### Principle

**Fig. 1:**
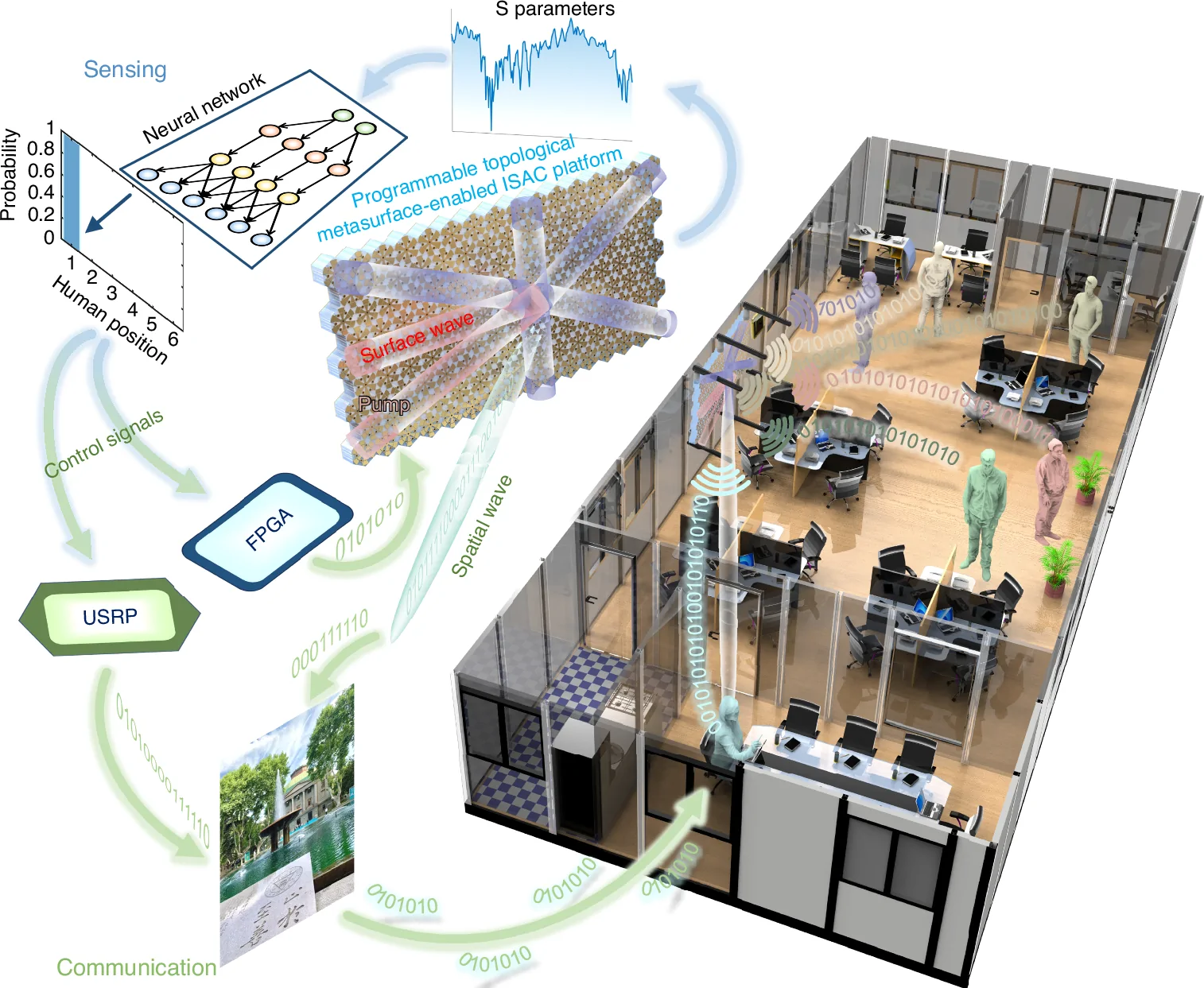
The schematic diagram of the programmable topological metasurface (PTM)-enabled integrated sensing and communication (ISAC) platform. An FPGA-controlled PTM is employed to manipulate surface wave propagation paths, enabling the collection of multidimensional EM data to train a neural network, hence realizing the human position sensing. Once the human position is recognized by the well-trained neural network, the PTM is reconfigured to regulate spatial waves, thereby enabling wireless communication. The nontrivial mode is realized by symmetry-broken valley-Hall domain-wall configurations for robust surface-wave sensing, whereas the trivial mode is realized by radiative phase-coding configurations for directional spatial-wave communication

As illustrated in Fig. 1, we introduce a PTM-driven ISAC system for human positioning and wireless communication. This system dynamically regulates the EM responses of four individual unit cells (Units 0, 1, 2, and 3) with distinct symmetries, enabling seamless transition between a topologically nontrivial valley-Hall state for protected surface-wave transport and a trivial radiative state for the conventional spatial-wave beamforming on the same PTM platform. This transition can effectively mitigate the mutual interference between surface and spatial waves. This sequential state-switching strategy is crucial for system integration. In particular, the sensing mode and communication mode require two distinct wave-control mechanisms—topological domain-wall transport of surface waves and radiative beamforming of spatial waves, respectively. Instead of forcing these mechanisms to operate simultaneously in a fixed state, the proposed PTM resolves the compatibility challenge by reconfiguring its unit-cell symmetry and coding state under the FPGA control. As a result, the same physical platform can firstly perform intelligent sensing and then, after the CNN-based position recognition, be reconfigured into a directional communication aperture without additional hardware. At the physical level, the FPGA-controlled biasing network reprograms the ON/OFF states of the embedded PIN diodes, thereby altering the microwave current distributions and effective symmetry of the unit cells. When the PTM is configured by the mirror-related C3-symmetry units, the inversion symmetry is broken, which lifts the Dirac degeneracy near the K/K’ valleys and opens a valley bandgap. As a result, the domains with opposite valley characteristics support nontrivial domain-wall edge states, allowing robust surface-wave transport for wireless sensing. By contrast, when the PTM is reconfigured into the radiative mode, the metasurface no longer relies on the domain-wall topological transport, but functions as a 1-bit phase-coded aperture that manipulates the reflection phase of spatial waves for far-field beam steering. In this way, FPGA directly switches not only the coding pattern but also the underlying EM mechanisms, namely from the topological surface-wave guidance to trivial radiative wavefront controls.

By leveraging the topological properties of PTM, the system facilitates robust and reconfigurable EM wave manipulations, thereby enhancing the versatility and efficiency of ISAC applications. Leveraging the quantum valley-Hall effect, PTM can dynamically control the surface waves along engineered domain-wall interfaces constructed by Units 2 and 3 with C3 symmetry (see Supplementary Notes1 and 2), thereby establishing topologically protected propagation pathways that are inherently robust against the structural defects and backscattering. Six high‑performance Vivaldi antennas are connected with coaxial lines welded at the terminus of each pathway to transduce the guided surface waves into radiated fields. The localized radiation fields excited by these domain‑wall interfaces have strong interactions with the passing humans, generating unique spectral characteristics that enrich the perceptual EM dataset for human localization. Then, the collected multidimensional EM datasets are used to train a dedicated CNN model for accurate classification of the human positions. Following successful position recognition, the PTM-enabled ISAC system adaptively switches to a topologically trivial radiative state, facilitating wireless communication. Capitalizing on the 1-bit phase modulation capability of Units 0 and 1 with C6 symmetry, the FPGA dynamically reconfigures the PTM into a spatial-wave control mode (see Supplementary Note 3). In this mode, directional wireless communication links are established using a Universal Software Radio Peripheral (USRP), enabling efficient information transmission towards the identified human location without requiring additional hardware modules. In practice, the state transition is implemented through FPGA-controlled bias reconfiguration of the embedded PIN diodes. The effective response time of the full ISAC platform is therefore determined not only by the PTM switching itself, but also by the overall sensing-and-control chain, including spectral-data acquisition, CNN inference, FPGA command updating, and communication triggering. While the present work is focused on demonstrating the feasibility of this switching-enabled workflow, a dedicated quantitative optimization of end-to-end latency will be an important step for future real-time deployment.

### Non-trivial valley-Hall PTM for intelligent human positioning

**Fig. 2:**
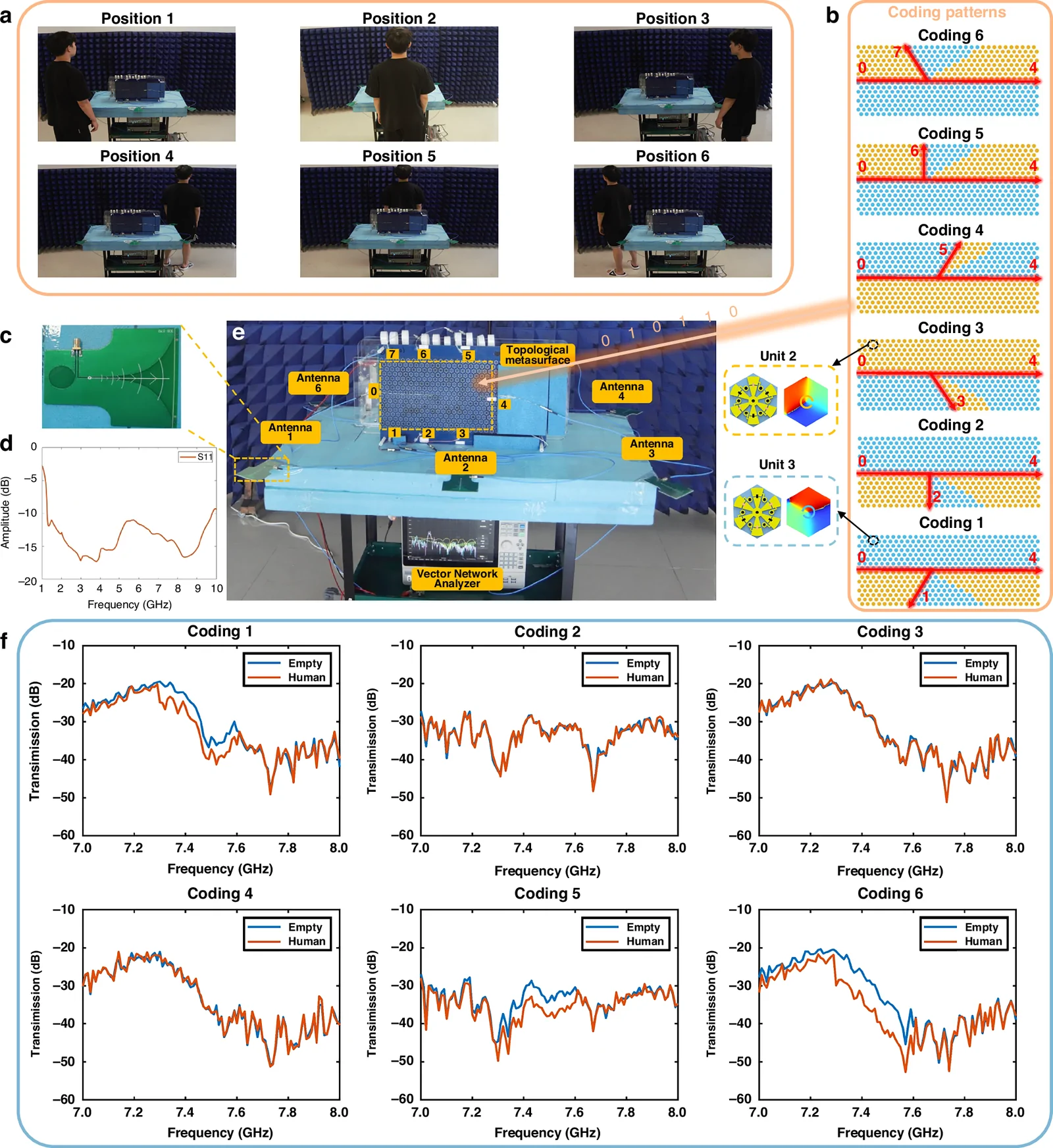
Demonstration of an intelligent positioning system based on programmable topological propagation routing. **a** Schematic representation of various detection areas centered around the programmable topological PTM. **b** Illustration of seven distinct propagation routing coding patterns, including topological straight waveguides and waveguides with bends at angles of ${60}^{^\circ }$, ${90}^{^\circ }$, and ${120}^{^\circ }$. In the one-to-two routing scheme, each coding pattern splits a single input channel into a straight reference branch connected to the VNA and a bent sensing branch connected to one of six fixed Vivaldi antennas. **c**, **d** The Vivaldi antenna utilized for sensing and its corresponding reflection coefficient ${S}_{11}$ magnitude frequency characteristics. **e** Photograph of the experimental setup for the intelligent positioning system based on programmable topological propagation routing, featuring the programmable topological PTM, multiple Vivaldi antennas, and a VNA. **f** Transmission characteristics of the straight waveguide routes at the detected object located at position 6 under different coding schemes, compared to the baseline non-human state (“Empty”)

Firstly, we explore the application of a PTM platform in intelligent human positioning, achieved through the dynamic control of the topological properties of spatial dimensions, as illustrated in Fig. 2. The PTM facilitates real-time switching of the states of embedded PIN diodes (the OFF state denoted as “0” and the ON state as “1”), enabling the realization of various topological edge states (see Supplementary Notes4 and 5). This flexibility not only allows for the construction of diverse and non-trivial domain-wall interfaces but also endows these interfaces with unique physical properties, including unidirectional propagation characteristics with valley chirality locking and significant robustness (see Supplementary Note 6). Six Vivaldi antennas are connected to six bent-branch terminals of the programmable one-to-two topological routing network, while the straight reference branch is connected to the VNA for the transmission measurement. The localized EM fields generated by the Vivaldi antenna can effectively interact with passing objects. The EM interaction produces unique spectral responses corresponding to different human positions along the same reception path, thereby providing richer and more reliable perceptual information for precise positioning. To validate the exceptional positioning performance of the proposed PTM, we divided the detection area surrounding the PTM into six equally sized square regions, as shown in Fig. 2a. Based on the aforementioned properties and the PTM’s flexible control over surface waves, we constructed six independently controllable one-to-two topological routing schemes on the PTM, as depicted in Fig. 2b. Here, “one-to-two” means that a single topological input channel is split into two output branches by a programmable domain-wall junction: a straight reference branch and a bent sensing branch. The straight branch is shared as the transmission-measurement path and is connected to two ports of the VNA, whereas the bent branch is terminated at a Vivaldi antenna to generate a localized radiative sensing node. Across the six FPGA programmed coding patterns, the bent branch is directed toward six fixed antenna terminals corresponding to upward and downward bends of 60°, 90° and 120°, respectively. This architecture enables the PTM to maintain stable reference transmission path while selectively activating different localized sensing branches for multidimensional human-position perception. The intelligent positioning system is equipped with six high-performance Vivaldi antennas (see Fig. 2c), whose broadband EM characteristics (see Fig. 2d) ensure accurate detection of localization information, as shown in Fig. 2e.

It should be noted that the six sensing channels are not excited simultaneously. Instead, the FPGA sequentially switches the six coding patterns, so that only one topological routing state is activated at a time during data acquisition. As a result, strict simultaneous inter-channel crosstalk is largely avoided by design. For the activated channel, the corresponding valley-Hall domain-wall mode strongly confines the guided surface-wave energy along the selected interface, while the topological transport further suppresses undesired leakage caused by backscattering, sharp bends, and moderate structural disorder. The guided energy is then converted into a localized radiative sensing field only at the addressed Vivaldi terminal. Therefore, the effective channel isolation in the present sensing scheme is originated from the combined action of time-multiplexed excitation, topological path confinement, and terminal-selective radiation.

When a target individual is positioned within the monitoring area near an activated Vivaldi terminal, the localized radiated field generated at the bent-branch end is coupled strongly to the nearby body, which in turn perturbs the transmission response of the corresponding topological routing network. These transmission variations are measured by the VNA and used as input features for the subsequent CNN-based position classification. Our findings indicate that different human body characteristics exhibit distinct transmission properties within specific frequency ranges. For instance, variations in the relative distance between the Vivaldi antenna and the target individual, as well as changes in their relative position within the monitoring area, lead to significant alterations in transmission characteristics. In order to accurately measure and collect these EM transmission parameters, we have designed an automatic data collection system using a high-performance VNA. Six volunteers aged 20–30 years with diverse body types are recruited to perform the intelligent human positioning system. An experimental volunteer stands at six distinct positions, with 500 rounds of EM data collected at each location. During each round, six different encoding patterns are sequentially switched, resulting in a comprehensive dataset of 111,000 entries (6 volunteers × 6 positions × 6 patterns × 500 rounds + empty × 6 patterns × 500 rounds) that capture the spatial and spectral variations associated with human positioning. The collected experimental datasets from different locations are demonstrated in Supplementary Note 7. The obtained data are subsequently employed to train a CNN to achieve efficient real-time localization based on neural network algorithms. It is noteworthy that we present only two segments of the transmission characteristics of the straight waveguide route under different coding patterns when the target individual is located at position 6, as shown in Fig. 2f. The results clearly indicate that significant changes in transmission characteristics, in response to the presence or absence of the target individual, are observed exclusively under coding pattern 6. In contrast, other coding patterns do not exhibit noticeable differences, underscoring the unique sensitivity of pattern 6 to the target-induced variations. This coding-selective spectral response qualitatively indicates limited effective crosstalk between adjacent programmed sensing routes, since the target-induced perturbation is predominantly captured by the matched topological channel rather than uniformly appearing across all coding states. This finding not only reveals the consistency between the exceptional robustness of the programmable topological PTM and the strong confinement properties of surface waves but also theoretically validates the feasibility of our proposed programmable topological PTM in intelligent positioning applications, showcasing its vast potential in future intelligent sensing technologies.

### CNN-based topological intelligent positioning

**Fig. 3:**
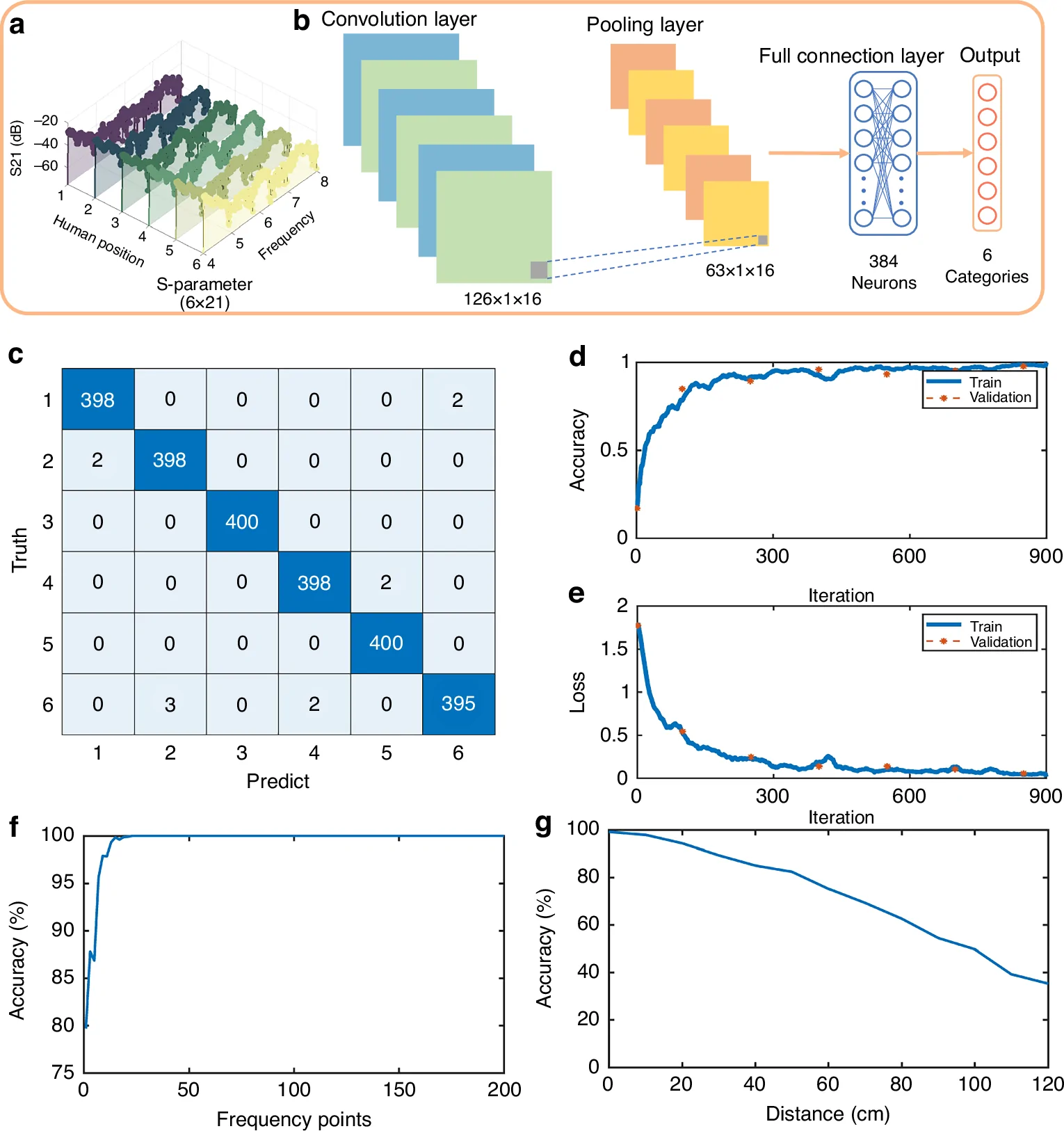
Network structure design and performance analysis of a CNN-based topological intelligent positioning. **a** Transmission parameter ${S}_{21}$ of the target individual measured at different positions during the experiments. **b** Schematic diagram of the CNN structure. **c** Confusion matrix of the test dataset, achieving an accuracy of 99.54%. Accuracy (**d**) and loss (**e**) curves during the training and validation process. **f** Performance of positioning recognition accuracy as the number of operational frequency points varies. **g** The positioning recognition accuracy is affected when the distance between a person’s position and the antenna’s position changes

Based on the unique feature information collected by PTM (see Fig. 3a), we implemented a data-driven positioning method. Motivated by the rapid development of machine-learning-enabled optimization and analysis in metamaterials and photonic systems^51–55^, CNNs are adopted here as efficient feature-extraction and classification tools for real-time human-position recognition. As powerful data analysis tools, CNNs can be trained on large-scale datasets to learn complex hierarchical representations for classification and clustering. The learned features can be used for target positioning, with CNNs primarily serving as the feature extractors. Compared to the traditional analytical methods, CNNs offer significant speed advantages. Once the initial training phase is completed, subsequent data classification and matching processes can be executed rapidly with minimal additional computational resources, which is particularly important in real-time applications. We divided the entire detection area into six sub-regions and used the CNN model to classify the receiving terminals into the corresponding sub-regions for precise localization. Given the positioning capabilities of PTM, the transmission parameters at different locations within each sub-region were labeled with the same tag. We then trained a CNN model using these labeled datasets, associating each input data with its corresponding class label. The network learns to map the original input data to these class labels by adjusting its parameters. The constructed CNN model, as shown in Fig. 3b, consists of a single convolutional layer. The input layer accepts the transmission parameters from 101 frequency points, and the output layer classifies the data into six positions (Positions 1–6). The convolutional layer in CNN captures local patterns using a 3 × 1 convolution kernel, enabling the network to learn the complex relationships and dependencies present in the experimental test data. The ReLU activation function introduces non-linearity into the network, allowing it to approximate complex functions that linear models cannot represent. Additionally, to ensure that the network captures both local and global features, a pooling layer further reduces the spatial dimensions of data. Finally, the output layer of network employs a softmax activation function to convert the extracted features into the classification predictions.

The CNN model is trained using the Stochastic Gradient Descent with Momentum (SGDM) optimizer, with a learning rate set to 1 × 10^−3^ and a batch size of 16. Here, we randomly selected 18,000 location data entries from a single volunteer to train the CNN model, in which 15,600 data are used as the training set and the remaining 2400 samples as the test set. The results of the training process are presented in Fig. 3d, e, showing the accuracy and loss curves, respectively. It is evident that the validation dataset achieved a classification accuracy of 99.54% after 900 iterations, with the confusion matrix after training depicted in Fig. 3c, demonstrating the high accuracy of the proposed topological intelligent positioning system and validating the feasibility of the CNN model. Based on the trained CNN model, we can accurately classify the receiving terminals into different sub-regions related to the human position, allowing for direct calculation of the corresponding coding patterns and the specific locations of targets. Furthermore, we analyzed the impact of various experimental parameters on recognition accuracy (see Supplementary Note 8). To further assess robustness beyond the controlled proof-of-concept setting, we additionally evaluated the sensing framework under cross-subject testing and perturbation-based non-ideal environments. Specifically, leave-one-subject-out validation across the six volunteers yielded an average localization accuracy of 97.23%, demonstrating favorable inter-subject generalization. We also introduced clutter-like and multipath-like perturbations with different severity levels to examine the stability of the learned topological spectral features. The results show that the recognition accuracy remains high under mild-to-moderate perturbations and decreases gradually only under stronger combined disturbances (see Supplementary Note 11). It should be noted that the present CNN-based positioning model is developed and validated in a controlled indoor environment, and therefore the current work does not claim full environment-invariant performance across arbitrary installation sites. Nevertheless, the collected dataset already includes six volunteers with diverse body types, posture variations, and multiple coding states, which introduces meaningful variability into the learned feature distribution. In addition, the supplementary 222-class classification result further indicates that the learned topological spectral features are not limited to a simple six-position labeling task, but retain substantial discriminability across different subjects, coding states, and unmanned conditions. From a deployment perspective, the 111,000-entry dataset should be regarded as a comprehensive research benchmark for model development rather than a required per-site retraining burden. In practical applications, a more realistic strategy would be to use the pretrained model as initialization and then perform site-specific calibration or fine-tuning with a smaller dataset when the new environment differs moderately from the original installation.

As shown in Fig. 3f, the distance between the target and Vivaldi antenna was 10 cm in experiments and the detection distance can be improved in the future by enhancing the transmission power. We further investigated the influence of varying the number of operational frequency points on accuracy. The results indicated that the recognition accuracy significantly decreased under the same network architecture as the frequency points are fewer than 10, a phenomenon attributed to the reduction in feature information. We also tested the recognition accuracy of target at varying distances from the Vivaldi antenna, as shown in Fig. 3g. When the distance increases, the recognition accuracy of human position decreases due to the energy attenuation during long-distance transmission, resulting in a significant reduction in data characteristics. Finally, we also trained the CNN model using the entire collected dataset of 111,000 samples to demonstrate the excellent performance of the model. The detailed information can be found in the Supplementary Note 10. It should be noted that the six discrete positions investigated in the present work are selected according to the current prototype configuration, in which six programmable sensing branches/terminals and six corresponding detection sub-regions are employed around the PTM. Therefore, six discrete positions should not be interpreted as a fundamental upper limit of the proposed positioning approach. In practice, the maximum number of distinguishable positions is jointly determined by the number of available programmable sensing channels, the degree of spatial confinement and overlap of the localized electromagnetic signatures between neighboring regions, the richness of the measured feature space (such as the number of effective frequency points and the signal strength), and the capability of the learning model. Consistently, our results show that reducing the number of frequency points or increasing the target distance degrades the recognition accuracy, indicating that feature dimensionality and signal attenuation directly influence the achievable spatial resolution. Moreover, the 222-class experiments in Supplementary Note 10 further suggest that the proposed PTM-based framework has meaningful scalability beyond the six-position proof-of-concept. A supplementary scalability analysis is provided in Supplementary Note 13, where the classification performance is evaluated as the number of distinguishable spatial positions increases within the current dataset. Additional benchmarking against the representative topological sensing and UWB-based human-sensing literature, together with discussions of mode purity/blind-spot considerations in the present PTM architecture, is provided in Supplementary Note 18.

### Trivial PTM for wireless communication system

**Fig. 4:**
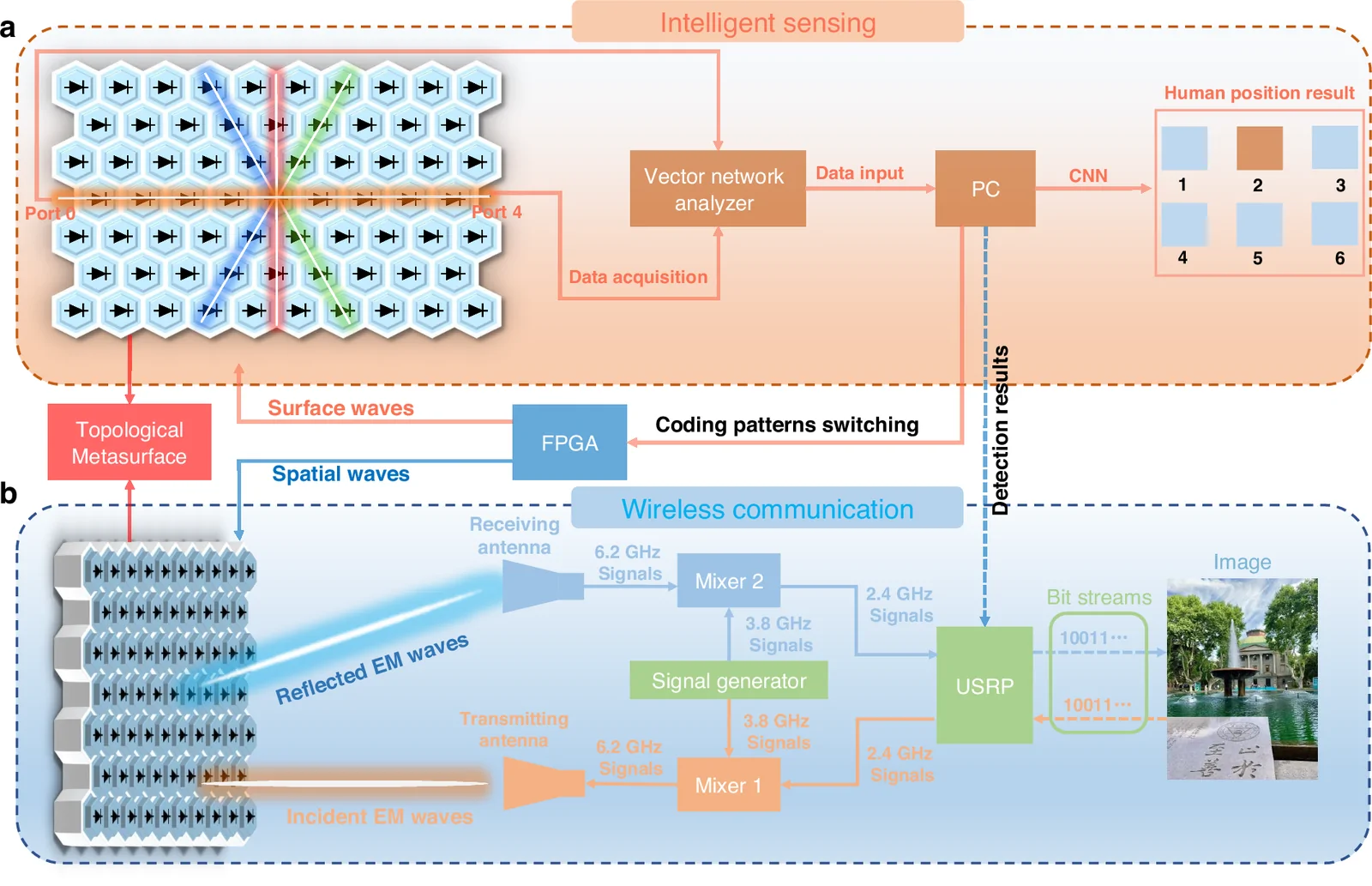
The detailed flowchart of the PTM-based ISAC system. **a** Schematic diagram of surface wave modulation for human position sensing based on the PTM. The multidimensional EM data corresponding to human positions are collected by a VNA and subsequently employed to train a specific CNN for high-precision localization. **b** Schematic of the PTM-enabled wireless communication system. Spatial-wave modulation mode is achieved by dynamically reprogramming the metasurface, and a USRP handles real-time signal modulation and demodulation for directional link establishment

The intelligent sensing results can provide compelling evidence for the feasibility of the proposed PTM‑based ISAC architecture, as schematically illustrated in Fig. 4. In the intelligent sensing stage of Fig. 4a, a VNA acquires the real‑time EM spectral response, which is then transferred to a personal computer (PC) for classification by a well‑trained CNN model. Upon determining the volunteer’s position, the localization result is immediately communicated to an FPGA‑based controller, which reconfigures the PTM to modulate spatial waves and establish a directed wireless communication link. In practice, the communication module is enabled only after the PTM reconfiguration is completed and a short guard interval is reserved, so that any residual transient mixed-mode leakage during the switching window does not overlap with payload communication. In the present prototype, this sensing-to-communication loop is implemented using a benchtop instrumentation chain consisting of a VNA, a PC, an FPGA controller, and a USRP. Therefore, the practical end-to-end response time is jointly determined by spectral acquisition, data transfer, CNN inference, FPGA control updating, PTM state reconfiguration, and communication triggering. The current work is focused on validating the feasibility of the switching-enabled closed-loop ISAC workflow, rather than providing a dedicated latency-optimized implementation. A quantitative characterization of the total cycle time will be an important direction for future engineering development. This sensing-to-communication feedback loop demonstrates the feasibility of a closed-loop ISAC workflow in dynamic scenarios. To further demonstrate the performance of the proposed PTM-based ISAC system, we subsequently demonstrated the functionality of wireless communication. The directional beams with different directions can be generated to construct wireless communication links when the embedded PIN diodes of the PTM are switched as the desired spatial encoding patterns by an FPGA (see Supplementary Note 3). In the present prototype, the demonstrated beam directions are determined by the selected coding periods of the one-bit phase-gradient aperture; thus, the current angular range is design-dependent rather than fundamental, and can be further extended in future implementations by reducing the coding period or adopting more advanced phase-control schemes. The entire PTM-based wireless communication system is exhibited in Fig. 4b, including a USRP, a PC, the PTM, transmitting and receiving antennas, two mixers and two signal generators. In the transmitting part, the transmitting image is modulated by a USRP with a 2.4 GHz tone signal and the modulated signal is then upconverted to the working frequency of the PTM (6.2 GHz) through a mixer supported with a signal generator of 3.8 GHz, which is sent to the transmitting antenna to excite the PTM. The EM waves carrying the information are transmitted to the specific receiver regulated by the PTM. In the receiving part, the corresponding information received by the receiving antenna is down-converted to a working frequency of 2.4 GHz by a mixer. The received information is finally demodulated and recovered as the transmitting image by a USRP. The wireless communication system is constructed by a USRP platform (NI USRP 2974) based on orthogonal frequency-division multiplexing (OFDM) signals with quadrature phase-shift keying (QPSK) modulation.

**Fig. 5:**
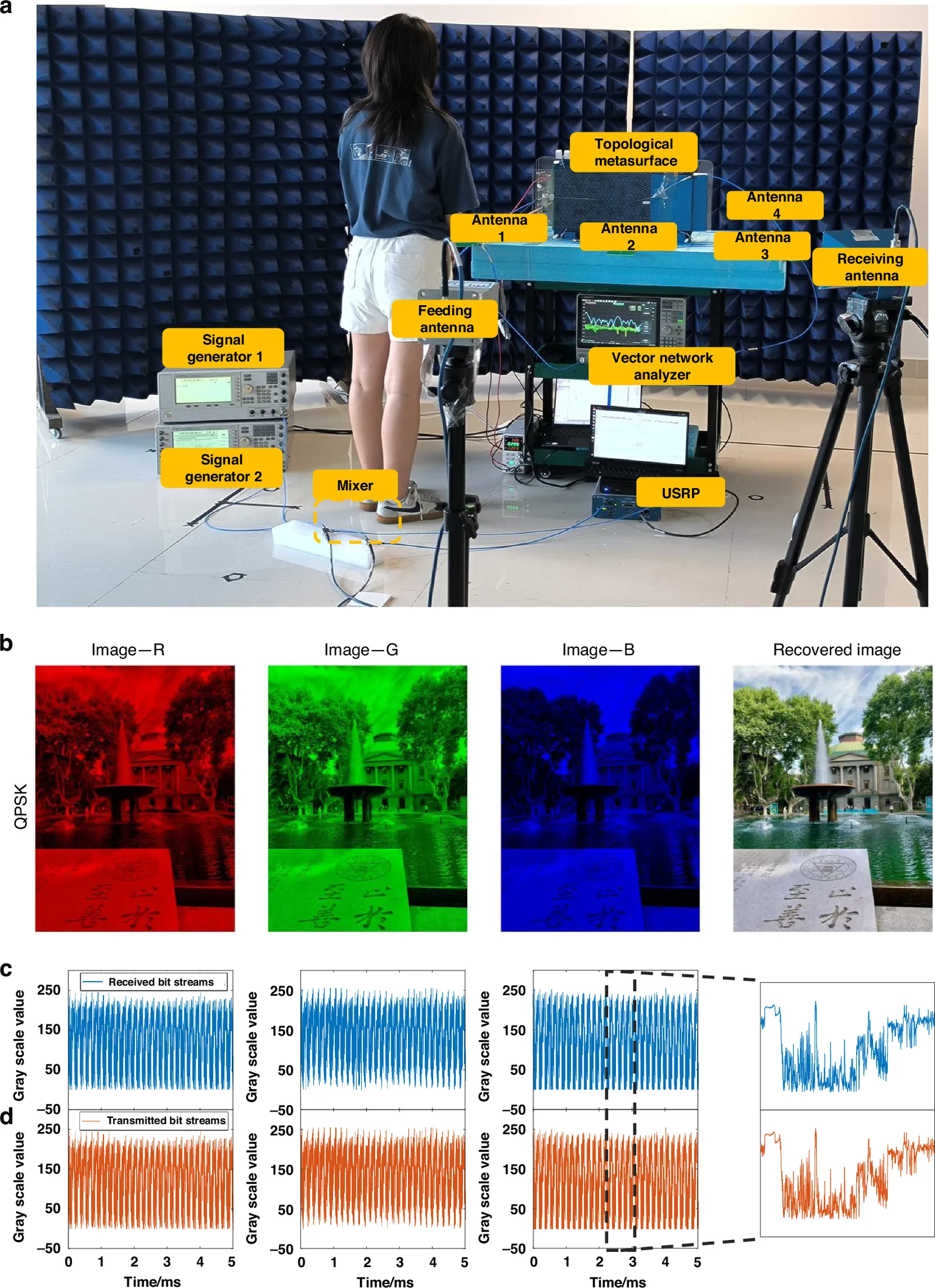
Experimental scenario of the PTM-based wireless communication. **a** The PTM-based wireless communication system platform. **b** The individual monochrome and synthesized full-color images from the received data. **c** The received bit streams in a PTM-based wireless communication system. **d** The transmitted bit streams in a PTM-based wireless communication system

The detailed experimental scenario of the proposed PTM-based ISAC platform is illustrated in Fig. 5a. The PTM prototype composed of 488 elements is supported by a multi-layer acrylic bracket, with the control circuit mounted on the rear side to control the PIN diode states of individual unit cells. To harvest energy propagating through distinct topological channels, eight coaxial lines are welded to the corresponding unit cells. Six coaxial lines are respectively connected with high‑performance Vivaldi antennas, transducing guided surface‑wave energy into radiated fields for high‑resolution human position sensing. The remaining two ports (straight topological path) are directly connected to a VNA for collecting EM data. In addition, a feeding horn antenna is connected with one port of a USRP via a mixer, which is used to illuminate the PTM. A receiving antenna, connected with the USRP’s second port via another mixer, is located at a designated spatial direction to receive EM waves carrying the information. The integrated ISAC system can be implemented by the proposed PTM. Upon a volunteer’s entry into a predefined spatial zone, the PTM can be rapidly programmed by an FPGA, sequentially switching six unique topological coding schemes to establish corresponding quantum valley‑Hall protected surface‑wave channels. At the terminus of each channel, high‑performance Vivaldi antennas transduce the guided surface waves into radiated fields, capturing multidimensional EM signatures that reflect the subject’s interaction with the localized EM fields. The multidimensional data is then fed into a well-trained CNN in real-time, achieving robust human‑position classification with an accuracy of 99.54%. Once the human position is identified by the CNN model, the PTM will be reconfigured with the corresponding spatial coding pattern through the FPGA and the USRP is triggered to modulate the baseband signals, constructing the specific wireless communication link in the spatial domain. The EM waves carrying information are modulated by the PTM and reflected to the receiving horn antenna located at a specific direction, ensuring that the corresponding information is demodulated and recovered (detailed information can be found in the Supplementary Note 9). In the wireless communication experiment, a full-color image is successfully transmitted to the receiver by simultaneously sending three monochrome images: Image-R, Image-G, and Image-B, as demonstrated in Fig. 5b. The received information is demodulated as bit streams in Fig. 5c. The received bit streams are then compared with the original transmitted bit streams of Fig. 5d, directly evaluating the performance of wireless communication systems. For a communication rate of 1 Mbps using the QPSK modulation scheme, the PTM‑based wireless communication system exhibits a bit error rate (BER) of ~4.2 × 10^−3^, thereby validating the proposed ISAC platform’s capability for robust wireless communication and high‑precision EM sensing.

From an architectural perspective, the main advantage of the proposed PTM-based ISAC platform lies in the reuse of a single programmable aperture and the reduction of duplicated sensing/communication hardware blocks. It should be noted that the present prototype is implemented as a benchtop validation system using a VNA, a PC, an FPGA controller, and a USRP, where the VNA serves as a laboratory sensing/data-acquisition instrument rather than a deployment-oriented integrated sensing receiver. Therefore, the hardware-efficiency benefit of the PTM concept should be assessed primarily at the architecture level, rather than directly inferred from the full laboratory instrument power budget. In addition, the present 1-bit radiative control is prioritized due to the simplified biasing and rapid reconfiguration, while the future higher-bit implementations may further improve the beamforming efficiency and SNR performance.

## Discussion

In this work, we demonstrated a PTM-enabled ISAC platform capable of human positioning and wireless communication through programmable transitions between non-trivial topological surface-wave states and trivial radiative states. By dynamically reconfiguring the electromagnetic response of its unit cells, the PTM integrates surface-wave and spatial-wave functionalities within a single physical structure. In its non-trivial valley-Hall phase, the PTM supports topologically protected domain-wall propagation, enabling robust near-field sensing in which localized electromagnetic fields interact strongly with passing human bodies and produce distinctive spectral signatures. These multidimensional spectral datasets train a CNN model that achieves human-position recognition accuracies exceeding 99%. Moreover, the 222-class supplementary results suggest that the underlying feature space supports richer discrimination than the six-position proof-of-concept, motivating future extension toward finer-grained spatial localization. Once the position is identified, the PTM is switched to its trivial radiative phase to generate directional spatial-wave beams for wireless communication, completing the dual-mode ISAC operation without additional hardware. Experimental and numerical results consistently validate the feasibility and effectiveness of this trivial–nontrivial topological switching framework. In practical deployment, the comprehensive 111,000-entry dataset established in this work should be viewed as a benchmark for model development rather than a mandatory retraining requirement for every new site. Future extensions may instead leverage the pretrained models together with reduced site-specific calibration and domain-adaptive learning strategies.

Beyond demonstrating dual functionalities, this work also highlights the key system-level challenge of combining the topological sensing routes and radiative communication states within one programmable architecture, and shows that such a challenge can be effectively resolved through sequential trivial-nontrivial state switching under FPGA control. Future work may further optimize the switching latency, environmental robustness, and large-scale system integration of this closed-loop PTM-enabled sensing-communication framework. While the present work establishes the physical feasibility and system-level effectiveness of the PTM-enabled ISAC, practical deployment in real-world environments will require further investigation under more complex EM conditions. We thus provide additional robustness evaluations based on cross-subject testing and perturbation-based environmental analysis, which partially examine the influence of the background clutter and multipath-like interference on sensing performance. The results indicate that the PTM-enabled framework preserves high recognition accuracy under mild-to-moderate perturbations. At the same time, simultaneous multiple-target sensing remains beyond the current experimental scope and is identified as an important direction for the future research. Future work will further quantify and optimize the end-to-end response speed of the platform, including PTM switching latency, control synchronization, and processing overhead, to better support real-time ISAC deployments in more practical scenarios. Overall, this study elevates programmable topological matter from component-level waveguiding to system-level functionality, highlighting topological phase transitions as a powerful mechanism for physical-layer switching in future 6 G, smart-environment, and healthcare sensing–communication platforms.

## Materials and methods

### Sample dataset collection process

The PTM is mounted vertically on a test platform, in which eight coaxial cables are welded to eight predefined topological routing terminals. Among them, two coaxial ports correspond to the shared straight reference branch and are connected to the two ports of the VNA, while the other six coaxial ports correspond to six bent sensing-branch terminals and are connected to six fixed Vivaldi antennas, representing the upward/downward branches with bending angles of 60°, 90° and 120°, respectively. The input and output of a specific straight propagation path were connected to the ports of a VNA (Agilent N5230C) to measure EM transmission parameters within the 4–8 GHz frequency band, which aligns with the bandgap frequency range of the supercell containing a domain-wall interface of the PTM. A computer was connected to the VNA through a network port and controls the automatic collection process of the sample dataset. Six volunteers were recruited to participate in the intelligent positioning system evaluation, including four males and two females aged between 20 and 30 years. The participants exhibited diverse body types to ensure the robustness of the experimental results. During each measurement session, a volunteer stood sequentially at six designated locations around the PTM to collect data, with 500 rounds of EM data acquired at each position. To increase the variability and diversity of the sample dataset, volunteers were allowed to freely swing their bodies and adopt different postures during data collection at each location. In order to introduce natural variability and enhance dataset robustness, six different encoding patterns were switched using an FPGA in each round to construct six topological propagation paths. These six coding states were applied sequentially rather than simultaneously, so the sensing dataset was collected from six time-separated channel responses instead of concurrently excited channels. The entire data acquisition process generated a substantial sample dataset comprising a total of 111,000 entries, of which 108,000 entries (6 volunteers × 6 positions × 6 patterns × 500 rounds) were collected in a manned state and the remaining 3000 entries (6 patterns × 500 rounds) were collected in an unmanned state. The 111,000 entries reported in this work correspond to the offline dataset collected for model training and validation. During online operation, the trained CNN performs inference on a single measured feature input from the current sensing state, rather than processing the full dataset in real time. For each measurement location, 18,000 data entries were collected from 6 different volunteers. Each volunteer contributed data over 500 rounds, with six distinct coding patterns switched during each round of data acquisition. This comprehensive sample dataset ensured a high degree of diversity and richness in the dataset, thereby providing a robust foundation for subsequent analysis. During the closed-loop operation, we remark that the sensing-to-communication transition is executed in a gated sequence: the spectral acquisition and classification are firstly completed, followed by the FPGA-driven PTM reconfiguration, and finally the USRP payload transmission is enabled. In the present proof-of-concept prototype, the FPGA is used to sequentially update the coding states during sensing and to reconfigure the PTM for communication after the position recognition. The current study is focused on the functional verification, and a dedicated measurement of end-to-end switching latency is left for future system-level optimization.

### Neural network design

The CNN model has been meticulously constructed to ensure effective feature extraction and accurate classification. The input layer utilizes a configuration capable of receiving multidimensional arrays with a dimension of 126 (6×21), which comprises data from 6 distinct positions across 21 frequency points. This structure is intentionally crafted to preserve both spatial and spectral information, thereby enhancing the model’s capability for effective feature learning. Subsequently, a convolutional layer is employed, utilizing a kernel size of 3×1 with 16 output channels, while maintaining consistent padding to preserve the spatial dimensions of the input and output. Following this, a rectified linear unit is introduced as the activation function, which effectively addresses non-linear problems and accelerates model convergence. After feature extraction, the network incorporates a pooling layer for down-sampling, using a 2×1 pooling kernel with a stride of 2. This process aims to reduce the size of the feature maps while retaining essential characteristics, thereby aiding subsequent learning and recognition by the model. Additionally, the network includes two fully connected layers, each comprising 384 neurons, to capture higher-level feature representations and ensure robust performance in complex tasks. The output layer consists of a fully connected layer designed to correspond to 6 target categories, integrating probability output and classification layers to achieve accurate predictions for the input data. During the training phase, we adopted stochastic gradient descent (SGD) as the optimization algorithm. The maximum number of iterations is set to 4, with an initial learning rate of 0.001, employing a dynamic learning rate adjustment strategy (with a decay factor of 0.9 and adjustment frequency of 10) to optimize the learning process. Moreover, we implement a mini-batch approach, with a batch size of 16, effectively balancing computational efficiency and memory usage. The data is randomly shuffled in each training epoch to improve the model’s generalization ability and mitigate the risk of overfitting. Regarding dataset partitioning, we randomly selected 15,600 samples for the training set, while the remaining 2400 samples were reserved for testing. This partitioning strategy ensures the independence of training and testing data, making the model evaluation more reliable and providing a robust basis for the model’s adaptability to new data.

## Supplementary information

Supplementary Information for Trivial-Nontrivial Programmable Topological Metasurfaces for Sensing and Communication

## Data Availability

The data that support the findings of this study are available from the corresponding author upon request.

## Code availability

The code that supports the findings of this study are available from the corresponding author upon reasonable request.
