# Supplementary material for: Trivial-nontrivial programmable topological metasurfaces for sensing and communication
Source: Light Sci Appl. 2026 Aug 20;15:354. doi: 10.1038/s41377-026-02419-x (PMC13494024; doi:10.1038/s41377-026-02419-x)
Supplement: Supplementary file 1 [file 41377_2026_2419_MOESM1_ESM.docx]

Supplementary Information for

Trivial-Nontrivial Programmable Topological Metasurfaces for Sensing and Communication

Qiang Xiao^1, 2, 3, †^, Long Chen^1, 2, †^, Qian Ma^1, 2, †,^ *, Yu Ming Ning^1, 2^, Zi Xuan Cai^1, 2^, Yi Zhang^1, 2^, Ze Gu^1, 2^, Jian Wei You^1, 2,^ *, Din Ping Tsai^3, 4, 5,^ *, Tie Jun Cui^1, 2,^ *

1. State Key Laboratory of Millimeter Wave, Southeast University, Nanjing 210096, China

2. Institute of Electromagnetic Space, Southeast University, Nanjing 210096, China

3. Department of Electrical Engineering and State Key Laboratory of Optical Quantum Materials, City University of Hong Kong, Kowloon, Hong Kong SAR 999077, China

4. State Key Laboratory of Terahertz and Millimeter Waves, City University of Hong Kong, Hong Kong, 999077, China.

5. Department of Physics, City University of Hong Kong, Kowloon, Hong Kong SAR 999077, China

*E-mail: [maqian@seu.edu.cn](mailto:maqian@seu.edu.cn), [jvyou@seu.edu.cn](mailto:jvyou@seu.edu.cn), dptsai@cityu.edu.hk, [tjcui@seu.edu.cn](mailto:tjcui@seu.edu.cn)

†These authors contributed equally to this work

**This ﬁle includes:**

Supplementary Notes 1-18

Supplementary Figures 1-29

Supplementary Equations 1-10

Supplementary References

**Supplementary Note 1: Fundamental physics of the topological valley-Hall metasurface**

This section discusses the fundamental physical properties of topological valley-Hall insulators (PTIs). Topological insulators have garnered significant attention due to their unique topological characteristics, which confer distinct electronic transport properties and offer various potential applications. Unlike photon Chern insulators and time-reversal symmetric spin-Hall topological insulators^1-3^, valley-Hall topological insulators^4-6^ require the breaking of inversion symmetry (P)^7^, leading to markedly different physical properties.

In a photonic crystal with two Dirac cones, external perturbations that break the P symmetry can induce a bandgap in the $K/{K^{'}}$ valleys, signaling the occurrence of a topological phase transition. The energy band structure can be described by a renormalized Dirac equation, where the mass term 𝑚 is proportional to the bandwidth, and its sign is determined by the non-zero local Berry flux in the $K/{K^{'}}$ valleys. The local Berry curvature is defined as:

$$\begin{aligned} \Omega_{n}\left( k \right)=\frac{\partial A_{y}}{\partial k_{x}}-\frac{\partial A_{x}}{\partial k_{y}}\#\left( S1 \right) \end{aligned}$$

Here, $A_{n}=-i\left\langle u_{n} | \partial_{k} | u_{n} \right\rangle$ represents the Berry connection associated with the periodic part of the Bloch wave function of the nth band. The valley Chern number, a topological invariant, is calculated by integrating the local Berry curvature around the valley and approximates to $\frac{1}{2}$^8^ under small perturbations. A key feature of valley-Hall topological insulators is the presence of gapless edge states, known as valley chiral states, at the interface between two valleys with opposite valley Chern numbers. These states exhibit opposite group and phase velocities in different valleys, demonstrating unique propagation characteristics. Due to valley-locked chirality, these valley chiral states can propagate even when the domain wall is bent at angles up to 120°, showcasing significant robustness against certain types of defects. Researchers have designed various unit cells that break P symmetry to achieve the functionality of valley-Hall topological insulators, typically by reducing the symmetry of triangular or honeycomb lattices to $C_{3}$.

**Supplementary Note 2: Reconfigurable topological light propagation**

We present a programmable topological metasurface that facilitates valley Hall plasmonic modes between two plasmonic crystals (D1 and D2), as illustrated in Fig. S1a. The unit cells of these crystals are distinctly marked in yellow and blue. D1 and D2 exhibit mirror symmetry about the x-axis, leading to identical band structures and a common bandgap, which underpins their propagation characteristics. This design enables the locked valley plasmonic modes along the pre-defined domain wall interfaces, promoting efficient information transmissions.

**Fig. S1 | Experimental results of programmable topological propagation paths. a** The topological metasurface composed of mirror-symmetric domains D1 and D2**. b** Layout of the near field scanning experimental setup**. c** The simulated results of the electric field distribution $\left| E_{z} \right|$ at 7.2 GHz for one‑to‑two topological waveguide splits with 60°, 90°, and 120° bends (up and down), respectively. **d** The measured results of the electric field distribution $\left| E_{z} \right|$ at 7.2 GHz for one‑to‑two topological waveguide splits with 60°, 90°, and 120° bends (up and down), respectively.

Additionally, we conducted an experimental investigation and numerical simulation into the metasurface’s resistance to sharp bends. As shown in Fig. S1b, the topological metasurface employs an FPGA-controlled network for dynamic modulation, allowing real-time adjustments of propagation paths based on the corresponding coding patterns. To test backscattering immunity during propagation along steep bends, we encoded each cell with FPGA, programming various topological and non-topological paths.

To receive real-time external human position EM data, we have designed six one-to-two topological transmission paths, among which the straight topological path is used to connect the vector network analyzer to collect human position data. In each one-to-two routing configuration, one output branch remains as a straight reference path, whereas the other output branch is bent toward one of six fixed sensing terminals, which are connected to the six Vivaldi antennas in the experiment. Six coding patterns are used to produce one‑to‑two split waveguides featuring up and down bends of 60°, 90°, and 120°, respectively. Numerical simulations of these configurations are performed using the finite‑element software COMSOL Multiphysics to evaluate the electric field behavior across the different domain‑wall interfaces. All simulated near-field distribution of $\left| E_{z} \right|$ at 7.2 GHz are clearly shown in Fig. S1c, demonstrating favorable transmission characteristics. In all simulation results, it can be seen that there are always simulation results for straight transmission, while the other transmission routes exhibit bends at angles of 60°, 90°, and 120° in both upward and downward directions. In addition, we have measured the near-field distribution of $\left| E_{z} \right|$ using the near-field experimental apparatus for accurate data collection. As shown in Fig. S1d, the propagation characteristics remained largely unaffected under various sharp bending conditions, highlighting their excellent adaptability. Furthermore, a comparison between the simulated and measured transmissions demonstrates a strong consistency in multiple key parameters, which further validates the reliability of our model and experimental design. Results indicate that propagation characteristics remain largely unaffected under these conditions, showcasing excellent adaptability. Finally, we examined the effects of defects and disorder on propagation. Despite potential manufacturing imperfections or operational failures, the topological interface exhibited considerable robustness under disordered conditions, highlighting its practical application potential. These findings establish a solid foundation for the future utilization of topological metasurfaces in complex environments. These measured and simulated field distributions also provide qualitative evidence that, for each programmed coding state, the surface-wave energy remains predominantly confined to the selected topological route, with limited leakage to adjacent paths. This route selectivity is important for suppressing spatial crosstalk in the intelligent-positioning scheme.

**Supplementary Note 3: Spatial-wave modulation of topological valley-Hall metasurface**

To achieve integrated control of surface waves and spatial waves, we propose a general design and optimization method for programmable elements based on energy band theory, building upon our previous research^9^. Surface wave characteristics are examined via band diagrams, while spatial wave manipulation leverages multimode resonance features. Our method unifies intrinsic mode band diagrams with radiation mode resonance characteristics, using band theory as a pivotal bridge. The detailed unit cell structure is illustrated in Fig. S2a.

**Fig. S2 | Characteristics of programmable topological metasurfaces in spatial wave manipulation. a** Schematic diagram of the programmable unit**. b** Amplitude and phase responses of the reflection coefficients for two units under a one-bit coding scheme, where the solid line represents the amplitude response and the dashed line represents the phase response. **c** Two distinct periodic coding sequences: “000111...”, “00001111...” and “000000111111...”. **d** Comparison of the numerical simulation results and experimental measurements of the 2D far-field scattering in polar coordinates corresponding to the coding patterns in **c**.

We begin by calculating the characteristic modes of a unit with $C_{3}$ symmetry using the COMSOL Multiphysics finite element solver, focusing on specific points in the first Brillouin zone ($k=0$) to observe resonant characteristics under normal incidence of a plane wave. Further simulations of oblique incidence are conducted using CST Microwave Studio, revealing a resonant frequency of approximately 6.3 GHz. Band diagram analysis allows us to optimize this frequency for effective modulation of both surface and spatial waves. One-bit phase modulation is achieved by adjusting the resonant frequency. After optimization, a phase difference of approximately 140° is achieved between digital states 0 and 1 at around 6.2 GHz, as shown in Fig. S2b, demonstrating the effectiveness of our encoding element. This approach guides the rapid design and optimization of topological metasurfaces for multifunctional metasurface devices. Using a gradient phase distribution coding metasurface, spatial waves can be directed into beams with varying deflection angles when illuminated by a vertically incident TE-polarized plane wave. The far-field scattering pattern is expressed as:

$$\begin{aligned} E\left( \theta,\varphi\right)=\sum_{i=1}^{M} \sum_{j=1}^{N} A_{ij}e^{-j\varphi_{ij}}e^{-jkp\sin\theta\left[ \left( i-\frac{1}{2} \right)\cos\varphi+\left( j-\frac{1}{2} \right)\sin\varphi\right]}\#\left( S2 \right) \end{aligned}$$

where $k$ is the wave vector; $A_{ij}$ and $\varphi_{ij}$ are $M\times N$ matrices representing the reflection amplitude and phase response, respectively; $P$ is the period of the unit cell; and $\theta$ and $\varphi$ are the elevation and azimuth angles of the far-field scattering pattern. According to generalized Snell’s law, varying periodic lengths of coding sequences alters scattering patterns：

$$\begin{aligned} \theta=\sin^{-1} \left( \frac{\lambda}{\Gamma} \right)\#\left( S3 \right) \end{aligned}$$

To validate the spatial wave manipulation capability, we illuminate the topological metasurface with different periodic coding sequences using a TE-polarized plane wave, resulting in dual beams with varying splitting angles. Fig. S2c shows the coding sequences, and full-wave simulations are conducted in CST Microwave Studio. The TE-polarized plane wave is incident on the metasurface, with far-field monitoring at 6.2 GHz. Fig. S2d displays corresponding far-field scattering patterns, demonstrating that one-bit phase control results in dual beams at different angles. It should be noted that the angular range observed in Fig. S2 is not an inherent limitation of the programmable topological metasurface itself, but is mainly determined by the coding period $\Gamma$ used for spatial-wave modulation. For the present prototype, the unit-cell width is $p=15 mm$ and the operating frequency for beam steering is $6.2 GHz$, corresponding to a free-space wavelength of approximately $\lambda\approx48.4 mm$. Therefore, for a coding sequence with period $\Gamma=Np$, the theoretical beam angle follows $\theta=\sin^{-1} \left( \frac{48.4}{15N} \right)$. The three coding sequences used in Fig. S2c correspond to $N=6$, $8$, and $12$, yielding theoretical steering angles of approximately 32.5, 23.8, and 15.6, respectively, which are consistent with the measured and simulated results in Fig. S2d. Hence, the demonstrated angular range arises primarily from the specific coding periods selected in this proof-of-concept design. In principle, a broader angular range can be achieved by reducing the coding period, decreasing the unit-cell size, or employing higher-bit phase control, although the practical performance will also be influenced by the finite aperture, nonideal phase quantization, and efficiency degradation at larger steering angles. In a microwave anechoic chamber, we measure the far-field scattering pattern of the metasurface prototype. The sample is fixed on an FPGA-controlled platform, enabling 360-degree horizontal rotation. A linearly polarized horn antenna generates a TE-polarized quasi-plane wave, with a receiving antenna capturing copolarized far-field signals at approximately 10 meters distance. The high agreement between experimental measurements and numerical simulations confirms the capability of our programmable topological metasurfaces in spatial wave manipulation.

**Supplementary Note 4: Chern number calculation**

The Chern number^10-15^ is a crucial integer in topological physics. Taking the transverse magnetic (TM) mode as an example, the Chern number for the nth band can be computed by integrating the Berry curvature over the first Brillouin zone (BZ), as expressed by the following equation:

$$\begin{aligned} C^{(n)}&=\frac{1}{2\pi i}\text{∮}_{\partial BZ}\left\langle\mathbf{u}_{n,e,\mathbf{k}}\left| \nabla_{\mathbf{k}} \right|\mathbf{u}_{n,e,\mathbf{k}} \right\rangle\mathrm{dk}\#\left( S4 \right) \end{aligned}$$

where, $\mathbf{F}_{n}(\mathbf{k})$ denotes the Berry curvature, and the Berry connection $\mathbf{A}_{n}(\mathbf{k})$ can be defined as:

$$\begin{aligned} \left\{ \begin{aligned} \mathbf{A}_{n}(\mathbf{k})=-i\left\langle\mathbf{u}_{n,e,\mathbf{k}}\left| \nabla_{\mathbf{k}} \right|\mathbf{u}_{n,e,\mathbf{k}} \right\rangle\\ \left| \mathbf{u}_{n,e,\mathbf{k}}(\boldsymbol{\rho}) \right\rangle=\left| e^{i\mathbf{k}\cdot\boldsymbol{\rho}}\mathbf{E}_{n,e,\mathbf{k}}(\boldsymbol{\rho}) \right\rangle\end{aligned} \right.\#\left( S5 \right) \end{aligned}$$

where, $\mathbf{u}_{n,e,\mathbf{k}}$ is the normalized eigenstate and $\left| \mathbf{E}_{n,e,\mathbf{k}}(\boldsymbol{\rho}) \right\rangle$ is the eigenstate.

During numerical calculations, it is essential to appropriately discretize the first Brillouin zone. In Fig. S3a, a square first Brillouin zone is presented. Subsequently, the Chern number is computed using the following formula:

$$\begin{aligned} C^{(n)}&=\frac{1}{2\pi}\sum_{\mathbf{k}\in\mathrm{BZ}} \mathrm{Imln}\left[ U_{\mathbf{k}_{1}\to\mathbf{k}_{2}}^{(n)}U_{\mathbf{k}_{2}\to\mathbf{k}_{3}}^{(n)}U_{\mathbf{k}_{3}\to\mathbf{k}_{4}}^{(n)}U_{\mathbf{k}_{4}\to\mathbf{k}_{1}}^{(n)} \right]\#\left( S6 \right) \end{aligned}$$

To obtain the Chern number, we discretize the integration to a summation $\sum_{\mathbf{k}\in\mathrm{BZ}} F_{\mathbf{k}}^{(n)}\Delta S_{\mathbf{k}}$. For Brillouin zones of varying shapes, similar discretization formats remain applicable. We employ an equivalent Brillouin zone that retains the same area as the first Brillouin zone but is triangular in shape, with its edges aligned with the reciprocal lattice vectors.

**Fig. S3 | Computational scheme for the calculation of Chern numbers. a** A square-shaped first Brillouin zone**. b** Discretization of the first Brillouin zone, and the computational scheme for the Chern number.

In the cases where the bands become degenerate, the Chern number cannot be assigned to each band individually, rendering the aforementioned formula inapplicable. In such situations, these degenerate bands share a composite Chern number, which is associated with the multiplet states. The calculation can be expressed as:

$$\begin{aligned} C^{(n\oplus n+1\oplus\cdots\oplus n+N-1)}&=\frac{1}{2\pi i}\text{∮}_{\partial BZ}\left[ \begin{matrix} \ddots& \vdots& \ddots\\ \cdots& \left\langle\mathbf{u}_{n_{l},e,\mathbf{k}}\left| \nabla_{\mathbf{k}} \right|\mathbf{u}_{n_{m},e,\mathbf{k}} \right\rangle& \cdots\\ .^{\cdot} & \vdots& \ddots\end{matrix} \right]d\mathbf{k}\#\left( S7 \right) \end{aligned}$$

By applying the Stokes’ theorem, the composite Chern number can be reformulated,

$$\begin{aligned} C^{(n\oplus n+1\oplus\cdots\oplus n+N-1)}\frac{1}{2\pi}\int_{\mathrm{BZ}} \mathrm{Tr}\nabla_{\mathbf{k}}\times\mathbf{A}_{\mathbf{k}}^{(n\oplus n+1\oplus\cdots\oplus n+N-1)}dS_{\mathbf{k}}\#\left( S8 \right) \end{aligned}$$

In the discretized Brillouin zone, the Chern number can be represented as:

$$\begin{aligned} \begin{aligned} &C^{(n\oplus n+1\oplus\cdots\oplus n+N-1)} \\ &=\frac{1}{2\pi}\sum_{\mathrm{BZ}} \mathrm{Imln}\left\{ det\left[ \mathbf{U}_{\mathbf{k}_{1}\to\mathbf{k}_{2}}^{(n\oplus\cdots\oplus n+N-1)}\mathbf{U}_{\mathbf{k}_{2}\to\mathbf{k}_{3}}^{(n\oplus\cdots\oplus n+N-1)}\mathbf{U}_{\mathbf{k}_{3}\to\mathbf{k}_{4}}^{(n\oplus\cdots\oplus n+N-1)}\mathbf{U}_{\mathbf{k}_{4}\to\mathbf{k}_{1}}^{(n\oplus\cdots\oplus n+N-1)} \right] \right\} \end{aligned}\#\left( S9 \right) \end{aligned}$$

For the calculation of the Chern number in transverse electric (TE) modes, the derived formulas remain valid.

**Supplementary Note 5: Topological characterization of PTM lattice**

In this section, we explore the topological characteristics of our unit cells, focusing on the topologically protected valley-chiral unidirectional transmission and robust transport properties linked to the quantum valley Hall effect. As demonstrated in our previous work^21^, we designed a metallic pattern comprising three pairs of sectoral segments with optimized unequal radii and a central circle to achieve desired topological properties. Through in-plane rotational modulation, we observe transitions of chiral states at specific K (K’) valley points, indicative of a topological phase transition between mirror-symmetric units, as illustrated in Fig. S5. This transition aligns with the bulk-boundary correspondence principle, resulting in topological edge states at the domain wall interface of the symmetric lattices, enhancing our understanding of light propagation.

The reconfigurability of each lattice allows dynamic control over light propagation paths by reconstructing encoded patterns, increasing system versatility. Our microwave-frequency platform mitigates metallic losses and facilitates surface plasmonic phenomena, showing promising practical applications. We focus on the transverse electric (TE) mode, where the electric field is confined to the plane and propagates along the xoy plane, constrained in the z-direction by total internal reflection. When the insulator maintains inversion symmetry, with equal radii of sectoral patterns, the system exhibits C_6_ symmetry, leading to Dirac cones at K and K’ points (see Fig. S4). However, unequal radii reduce the symmetry from C_6_ to C_3_, breaking inversion symmetry and affecting the degeneracy of Dirac points, which results in a bandgap around the Dirac frequency.

Additionally, the lattice displays left-handed circularly polarized (LCP) or right-handed circularly polarized (RCP) modes in different bands, with opposite polarizations in the same band across different lattices, elucidating the emergence of valley Hall states. The lattice exhibits non-zero Berry curvature, with calculations showing that Berry curvatures are equal in magnitude but opposite in sign at K and K’ valleys, leading to a total curvature of zero for individual bands. Integrating the Berry curvature around the valleys yields a half-integer valley Chern number: +1/2 at K and -1/2 at K’, with the opposite for the other band. This difference in valley Chern numbers between the domain wall interfaces of the two lattices results in the existence of valley Hall edge states within the bandgap.

**Fig. S4 | Optical responses of the unit cell with** $\boldsymbol{C}_{\boldsymbol{6}}$ **point symmetry. a** Photonic band structure. Due to the C_6_ symmetry-inversion protection, two Dirac cones exist at the K point in the band diagram. The electric ﬁeld distributions of $\left| E_{z} \right|$ and the phase distributions of the degenerate eigenmodes. Due to the plasmon-induced ﬁeld conﬁnement effect, the electric ﬁeld is conﬁned around the outer arms. The eigenmodes at the points ① and ② have opposite chirality**. b** Photonic band structure. Due to the C_6_ symmetry-inversion protection, a Dirac cone exists at the K point. The electric ﬁeld distributions of $\left| E_{z} \right|$ and the phase distributions of degenerate eigenmodes. Due to the plasmon-induced ﬁeld conﬁnement effect, the electric ﬁeld is conﬁned around the inner arms as all diodes are switched off. The eigenmodes at the points ① and ② have opposite chirality.

**Fig. S5 | Optical responses of the unit cell with** $\boldsymbol{C}_{\boldsymbol{3}}$ **point symmetry. a** Photonic band structure. Due to the breaking of the C_6_ symmetry, the Dirac point is gapped out. The electric ﬁeld is conﬁned to the odd inner arms, because the bridge of microwave current is terminated when the corresponding diode is switched off. On the contrary, the electric ﬁeld is conﬁned to the even outer arms as the corresponding diodes are switched on. Moreover, the eigenmodes belonging to the ﬁrst and second band have opposite chirality**. b** Photonic band structure. Due to the breaking of the C_6_ symmetry, the Dirac point is gapped out. The electric ﬁeld is conﬁned to the even inner arms, because the bridge of microwave current is terminated when the corresponding diode is switched off. On the contrary, the electric ﬁeld is conﬁned to the odd outer arms as the corresponding diodes are switched on. The eigenmodes belonging to the ﬁrst and second band have opposite chirality.

**Supplementary Note 6: Projected bands of domain-wall interfaces and the unidirectional topological propagation feature**

To validate the topological properties of nontrivial domain wall interface modes, we encoded the unit cell to create two serrated valley-Hall domain wall interfaces^16,17^, labeled interface A and B in Fig. S6. These interfaces separate domains filled with digital units 1 and 2. By adjusting the length difference between the two arms, we can precisely tune the valley gap, as depicted in the projected band diagrams in Fig. S6. Each configuration reveals two types of valley Hall interface modes, with blue (red) solid lines indicating the nontrivial modes for interface A (B) and gray shaded regions representing projected bulk modes. Minimizing the valley gap helps distinguish it from slip-symmetric interfaces, but achieving a larger bulk topological band gap is crucial for maintaining the valley-Hall state’s robust topological properties. We carefully optimized parameters to balance a well-defined valley-Hall phase with a broad topological spectral range for enhanced device functionality.

**Fig. S6 | Domain-wall interface modes. a** Topological domain-wall interface. The interfaces A and B separate two crystal domains, which are filled with digital units 1 and 2, respectively**. b** Projected band diagrams. The topological bandgap increases with the lengths of outer arm.

**Fig. S7 | The unidirectional topological propagation feature.** Simulated unidirectional propagations with the RCP (**a**) and LCP (**b**) excitations. White scale bar, 30 mm.

**Fig. S8 | Simulated and measured domain-wall interface modes.**

To investigate the unidirectional propagation characteristics of the topological interface modes, we design a straight waveguide with a B-type interface and use a chiral source composed of six electric dipoles to excite right-handed circularly polarized (RCP) or left-handed circularly polarized (LCP) waves, with the adjacent dipoles having a phase difference of $\pm\pi/3$. At a source frequency of 6.7 GHz, two distinct waveguide modes with opposite group velocities were generated at points 1 and 2. Notably, the chirality of the interface mode at point 1 is opposite to that at point 2, enabling unidirectional microwave propagation through selective excitation of RCP or LCP vortex states, as validated numerically in Fig. S7. Experimental results further confirm a high consistency with the numerical simulations, validating the effectiveness and stability of our constructed platform, as shown in Fig. S8.

**Supplementary Note 7: Experimental dataset**

In this section, we present the transmission coefficient datasets obtained from platform tests conducted at six different locations, as shown in Fig. S9. Through careful analysis of these data, we observe significant variability in the transmission coefficients across different locations, reflecting the impact of various factors on signal transmission characteristics. To demonstrate the influence of the human body on surface waves at different positions, we clearly present the transmission coefficients for a person standing at positions 1 to 5 under six different coding schemes, as shown in Fig. S10-14. Specifically, the transmission coefficients at each location are influenced by multiple factors, including the local optical properties of the materials, variations in geometric structures, and fluctuations in environmental conditions.

The rich diversity of this dataset not only provides a crucial foundation for our subsequent analyses but also ensures the accuracy and effectiveness of the neural network learning process. By thoroughly learning from the diverse transmission coefficients, the neural network can better identify and classify different signal patterns, thereby enhancing the overall performance of the system. This process is vital for improving the reliability and precision of signal processing, as it enables the system to maintain efficient operation in complex environments. Moreover, the variability in the transmission coefficients offers valuable insights that contribute to a deeper understanding of the platform’s performance under different conditions.

**Fig. S9 | Experimental data set in different positions. a-f** Transmission parameter $S_{21}$ of the target individual measured at position 1 (**a**), position 2 (**b**), position 3 (**c**), position 4 (**d**), position 5 (**e**), and position 6 (**f**) during the experiments

**Fig. S10 | Transmission characteristics of the straight waveguide routes at the detected object located at position 1 under different coding schemes, compared to the baseline non-human state (“Empty”).**

**Fig. S11 | Transmission characteristics of the straight waveguide routes at the detected object located at position 2 under different coding schemes, compared to the baseline non-human state (“Empty”).**

**Fig. S12 | Transmission characteristics of the straight waveguide routes at the detected object located at position 3 under different coding schemes, compared to the baseline non-human state (“Empty”).**

**Fig. S13 | Transmission characteristics of the straight waveguide routes at the detected object located at position 4 under different coding schemes, compared to the baseline non-human state (“Empty”).**

**Fig. S14 | Transmission characteristics of the straight waveguide routes at the detected object located at position 5 under different coding schemes, compared to the baseline non-human state (“Empty”).**

**Supplementary Note 8: Effect of different parameters on perceptual categorization**

We further investigated the influence of two key parameters on the accuracy of perceptual classification, namely the learning rate and the maximum number of training epochs (max epochs). Firstly, regarding the impact of the learning rate, our experimental results indicated that as the learning rate gradually increased, the model’s classification accuracy exhibited a steady upward trend. This suggests that a higher learning rate can accelerate the model's learning of data features, thereby enhancing its classification performance. However, we observed a significant decline in accuracy when the learning rate reached 0.1, as shown in Fig. S15. This phenomenon may be attributed to issues such as overfitting or gradient explosion, which prevent the model from converging effectively. Therefore, after a systematic analysis and experimentation, we ultimately determined that the optimal learning rate should be set at 0.01, striking a balance between learning speed and model stability to achieve the best classification performance.

**Fig. S15 | The effect of learning rate on perceptual recognition accuracy.**

Secondly, we conducted an in-depth analysis of the maximum number of training epochs (max epochs). The results of Fig. S16 demonstrate that as the maximum number of epochs gradually increased, the classification accuracy also improved steadily and eventually stabilized. This indicates that an appropriate increase in the number of training epochs allows the model to thoroughly learn the underlying features present in the training data, thereby enhancing its generalization capability. However, an excessive number of epochs may lead to the risk of overfitting; hence, after conducting multiple experiments, we ultimately concluded that the maximum number of training epochs should be set to 5, ensuring that the model maintains high accuracy while avoiding overfitting.

**Fig. S16 | The effect of MaxEpochs on perceptual recognition accuracy.**

In summary, through meticulous tuning of the learning rate and the maximum number of training epochs, we were able to significantly enhance the accuracy of the model’s perceptual classification. These findings not only provide critical insights for subsequent model optimization but also lay a solid foundation for future research directions, further advancing the application of deep learning in perceptual and classification tasks.

**Supplementary Note 9: High-quality information transmission**

In this section, we provide a comprehensive demonstration of the robust information transmission capabilities^18-20^ based on topological characteristics. Through a series of experimental tests, we observed that the system exhibits outstanding quality in information transmission, with a bit error rate (BER) of about 4.2×10^-3^, which can be considered negligible. This result indicates the system’s ability to achieve high-quality information delivery at an extremely low error rate, demonstrating exceptional resistance to interference. To further demonstrate the reliability of our system, we conducted experiments at six different locations (see Fig. S2d), utilizing a neural network for training to establish fixed configurations for each position in order to execute information transmission tasks for various objects. We achieved precise target recognition at each location, successfully transmitting six distinct images to their respective users, as shown in Fig. S17. This process not only highlights the system’s effectiveness in diverse information transmission tasks but also showcases its adaptability and flexibility under varying environmental conditions.

In future research, we plan to further optimize the system’s algorithms and architecture to enhance its performance in even more complex scenarios. Specifically, we will explore the utilization of emerging deep learning techniques and advanced signal processing methods to improve transmission efficiency and information quality. Through this ongoing research and refinement, we anticipate expanding the system’s application scope, enabling it to play a significant role in cutting-edge technological fields such as smart cities, the Internet of Things (IoT), and autonomous driving. In summary, the findings presented in this section not only highlight the robust information transmission capabilities based on topological characteristics but also lay a solid foundation for future advancements. We believe that these innovative research efforts will contribute to the progress of information transmission technology and propel development in related fields.

**Fig. S17 | Image transfer at different locations.** Once the recognition results for the six locations are obtained, the PTM system adaptively performs wireless communication at each respective location to transmit different images.

**Supplementary Note 10: Perceptual analysis based on multi-objective data**

In this section, we will delve into the remarkable capabilities exhibited by our platform in the realm of perception classification. Through a meticulously designed experiment, we engaged six different subjects, each of whom operated under six distinct coding schemes while positioned in six separate locations for data collection. At each location, we gathered 500 sample data points, resulting in a total of 216 unique experimental scenarios. Additionally, to comprehensively assess the performance of the system, we incorporated data collection in an unmanned state, thereby increasing the total number of scenarios to 222, which effectively highlights the adaptability and flexibility of our platform.

**Fig. S18 | Confusion matrix for the 222 classifications.** Numbers 1-6 represent the transmission status of six groups of codes in unmanned conditions. Numbers 7-42, 43-78, 79-114, 115-150, 151-186, and 187-222 represent the transmission status of six testers in six groups of codes at six positions, respectively.

For the experiments, we employed the same deep learning neural network architecture utilized in the main text for feature extraction and classification. After training, we achieved an impressive classification accuracy of 98.57% across 21 characteristic frequency points, further substantiating the robust capabilities of our platform in perception classification tasks, as depicted in Fig. S18. It is noteworthy that we systematically encoded these 222 scenarios: numbers 1 to 6 represent the unmanned state under the six coding paths, while numbers 7 to 222 sequentially correspond to the six different subjects under the six coding paths across the six testing locations. Specifically, the encoding between numbers 7 and 42 is further subdivided: numbers 7 to 12 correspond to the six positions under coding path 1, numbers 13 to 18 correspond to coding path 2, and so forth, culminating in number 42 representing the six positions under coding path 6.

**Fig. S19 | The identification and classification results of the six test subjects with different codes and different positions.**

To provide a more comprehensive demonstration of our platform’s superior performance, we present in Figs. S19 and S20 the classification results for the same subject under different positions and varying coding schemes, as well as for different subjects at the same position with the same coding scheme. The experimental outcomes indicate that our system consistently exhibits high robustness and stability across diverse experimental conditions, maintaining a satisfactory level of recognition accuracy. These findings not only validate the efficacy of our platform but also provide significant theoretical support and practical reference for future research endeavors.

**Fig. S20 | Recognition and classification results for 36 different codes and different positions for unmanned and six different test subjects.**

**Supplementary Note 11: Robustness evaluation under non-ideal environments**

**Fig. S21 | Cross-subject generalization performance under leave-one-subject-out validation.** The CNN was trained on data from five volunteers and tested on the remaining unseen volunteer. The results show consistently high localization accuracy across all six subjects, indicating good inter-subject generalization of the learned topological spectral features.

**Fig. S22 | Robustness of the sensing framework under environmental perturbations.** Recognition accuracy is evaluated under clutter-like, multipath-like, and combined perturbations with increasing severity. The results indicate that the learned spectral features remain discriminative under mild-to-moderate perturbations, while stronger combined perturbations lead to a more noticeable reduction in accuracy.

**Table S1 | Summary of robustness evaluation.**

| **Evaluation setting** | **Accuracy (%)** |
| --- | --- |
| Baseline single-volunteer test | 99.54 |
| 222-class extended classification | 98.57 |
| LOSO mean ± std | 97.23 ± 0.44 |
| Mild clutter | 98.84 |
| Moderate clutter | 97.56 |
| Strong clutter | 95.91 |
| Mild multipath-like | 98.63 |
| Moderate multipath-like | 97.12 |
| Strong multipath-like | 95.47 |
| Mild combined | 98.21 |
| Moderate combined | 96.38 |
| Strong combined | 93.84 |

To further evaluate the sensing robustness of the proposed PTM-enabled intelligent positioning system beyond the controlled proof-of-concept setting, we carried out additional analyses from two perspectives (see Table S1). First, we examined the cross-subject generalization capability using a leave-one-subject-out (LOSO) validation protocol across the six volunteers, as shown in Fig. S21. In each trial, the CNN model was trained on the data from five volunteers and tested on the remaining unseen volunteer. The obtained accuracies for the six held-out subjects were 97.8%, 96.9%, 97.3%, 96.7%, 97.6%, and 97.1%, respectively, corresponding to an average accuracy of 97.23%. These results indicate that the learned topological spectral features exhibit favorable inter-subject generalization.

Second, we investigated robustness under non-ideal environmental perturbations (see Fig. S22). To emulate background clutter and reflection-induced distortions in a controlled manner, clutter-like, multipath-like, and combined perturbations with increasing severity were introduced during the evaluation stage. Under clutter-like perturbations, the localization accuracies were 98.84%, 97.56%, and 95.91% for mild, moderate, and strong conditions, respectively. Under multipath-like perturbations, the corresponding accuracies were 98.63%, 97.12%, and 95.47%. For the combined perturbation condition, the accuracies were 98.21%, 96.38%, and 93.84%, respectively. These results demonstrate that the proposed sensing framework maintains high recognition performance under mild-to-moderate environmental disturbances, while stronger combined perturbations lead to a gradual but expected degradation in classification accuracy.

**Supplementary Note 12: Spatial crosstalk and effective isolation between adjacent coding channels**

An important question for the intelligent-positioning scheme is whether adjacent programmed coding channels introduce substantial spatial crosstalk, which could degrade the classification accuracy. In the present system, the six sensing channels are not operated simultaneously; instead, the FPGA sequentially switches the six coding patterns during each measurement round. Therefore, the acquired sensing features correspond to six time-separated channel states, rather than six concurrently excited channels. This time-multiplexed operation already suppresses strict simultaneous inter-channel crosstalk at the system level.

For each activated coding state, the EM energy is guided primarily along the selected valley-Hall domain-wall interface. Owing to the topological confinement of the nontrivial state, the surface-wave propagation remains localized along the programmed route, even in the presence of bends and moderate disorder. The guided energy is finally converted into a localized radiative sensing field only at the addressed Vivaldi terminal. As a result, the effective isolation mechanism in the present platform arises from the combination of sequential excitation, route-selective topological transport, and terminal-selective radiation.

To provide a more intuitive assessment of this effect, we define a normalized channel-response matrix 𝑅, whose element $R_{ij}$ denotes the normalized spectral perturbation strength measured under coding channel 𝑗 when the target is located at sensing region 𝑖 (see Fig. S23). In an ideal fully isolated system, 𝑅 would be purely diagonal. In practice, weak off-diagonal responses may appear due to residual route-to-route leakage, nonideal field overlap, and measurement noise. The representative results show a dominant diagonal response together with much weaker off-diagonal components, indicating that the target-induced perturbation is predominantly captured by the matched channel. To further quantify this effect, an effective isolation metric is introduced as

$$\begin{aligned} I_{ij}=20\log_{10}\left( \frac{R_{ii}}{R_{ij}} \right),i\neq j,\#\left( S10 \right) \end{aligned}$$

where $R_{ii}$ is the matched-channel response and $R_{ij}$ is the response of a non-matched channel. Larger $I_{ij}$ indicates better suppression of spatial crosstalk. The results show that the first-adjacent channels already exhibit clear attenuation relative to the matched channel, while next-nearest and non-adjacent channels are further suppressed. These observations are consistent with the strong route confinement shown in Fig. S1 and help explain why the CNN can maintain high positioning accuracy. Although a fully systematic route-to-route isolation measurement matrix will be valuable in future work, the present analysis provides direct qualitative and semi-quantitative support that spatial crosstalk between adjacent programmed sensing channels remains limited in the current PTM-based sensing scheme.

**Fig. S23 | Effective channel isolation and spatial crosstalk analysis of the six programmed sensing channels. a** Normalized channel-response matrix, showing that the target-induced perturbation is strongest in the matched coding channel and much weaker in adjacent and non-adjacent channels. **b** Averaged effective isolation level for first-adjacent, second-adjacent, and non-adjacent channels, demonstrating progressive suppression of route-to-route leakage away from the matched channel.

**Supplementary Note 13: Scalability analysis of distinguishable positions**

The six-position classification presented in the main text corresponds to the current PTM prototype, which employs six programmable sensing branches and six predefined detection sub-regions. To further examine the extensibility of the proposed framework, we additionally evaluate how the positioning performance evolves as the number of distinguishable spatial classes increases within the current dataset. Specifically, the measured dataset is regrouped into classification tasks with $K=2,3,4,5$, and $6$ positions, and the same CNN architecture is retrained under the corresponding class definitions. The resulting classification accuracies are summarized in Fig. S24.

As expected, the recognition accuracy decreases gradually as the number of distinguishable positions increases, because finer spatial partitioning leads to stronger overlap among neighboring position-dependent spectral signatures. Nevertheless, the results show that the proposed PTM-based sensing framework maintains high classification performance across all tested values of K, indicating favorable scalability within the current prototype configuration.

To further understand the limiting factors, we calculate the pairwise spectral separability between the six positions by evaluating the normalized distance between the averaged feature vectors of different positions. The corresponding separability matrix is shown in Fig. S25. The results indicate that the practical upper limit on the number of distinguishable positions is governed not by a single factor, but jointly by the number of programmable sensing routes, the spatial confinement of localized electromagnetic fields, the overlap of neighboring spectral signatures, the number of effective frequency points, signal attenuation with distance, and the capacity of the learning model. These observations are also consistent with the results in the main text and Supplementary Note 8, where reduced frequency-point number or increased target distance leads to lower recognition accuracy, and with Supplementary Note 10, where the 222-class experiment further demonstrates that the underlying feature space is richer than a simple six-position proof-of-concept.

**Fig. S24 | Classification accuracy as a function of the number of distinguishable positions.** The measured dataset is regrouped into K=2,3,4,5, and 6 spatial classes, and the same CNN framework is retrained under each class definition. Error bars indicate the standard deviation over repeated random train/test splits.

**Fig. S25 | Pairwise spectral separability matrix of the six spatial positions.** Each matrix element represents the normalized distance between the averaged spectral feature vectors of two positions. Larger values indicate better spectral distinguishability.

**Supplementary Note 14: Implementation-level latency considerations of the closed-loop PTM-enabled ISAC system**

To further clarify the practical response characteristics of the proposed PTM-enabled ISAC platform, it is important to distinguish the intrinsic device-level switching speed from the end-to-end system-level operational latency.

In the present platform, the reconfiguration of the metasurface is realized through FPGA-controlled bias switching of the embedded PIN diodes. The intrinsic state transition of the PIN diodes occurs at the nanosecond scale, indicating that the physical switching of the PTM itself is not the dominant bottleneck of the present prototype. Meanwhile, the 111,000 spectral entries reported in the manuscript correspond to the offline dataset collected for CNN training and validation, rather than the amount of data processed in a single online inference event. During operation, the trained CNN processes only one measured feature input from the current sensing state and outputs one location label.

Accordingly, the practical end-to-end cycle of the present benchtop system is jointly determined by several stages: spectral acquisition by the VNA, data transfer to the host computer, CNN inference, FPGA control updating, PTM state reconfiguration, and communication triggering. Since the PIN-diode switching itself is at the nanosecond scale, the dominant overhead in the current proof-of-concept setup is expected to arise mainly from the instrumentation and control chain, rather than from the intrinsic physical reconfiguration of the metasurface. The latency budget can therefore be qualitatively expressed as

$$\begin{aligned} T_{\mathrm{total}}=T_{\mathrm{acq}}+T_{\mathrm{transfer}}+T_{\mathrm{CNN}}+T_{\mathrm{ctrl}}+T_{\mathrm{comm}}\#\left( S11 \right) \end{aligned}$$

where $T_{\mathrm{acq}}$ is the VNA-based spectral acquisition time, $T_{\mathrm{transfer}}$ is the host-side data transfer time, $T_{\mathrm{CNN}}$ is the single-sample inference time, $T_{\mathrm{ctrl}}$ is the FPGA updating and PTM reconfiguration overhead, and $T_{\mathrm{comm}}$ is the communication-link triggering time. Because the intrinsic PIN-diode switching time is much smaller than the other terms, it is absorbed into $T_{\mathrm{ctrl}}$ in this formulation. Table S2 summarizes the latency-related interpretation of the current system. These considerations indicate that the proposed work should be regarded as a switching-enabled closed-loop proof-of-concept, while future engineering optimization can further reduce the end-to-end cycle time through tighter hardware integration, faster acquisition electronics, and on-board/edge inference.

**Table S2 | Representative implementation-level latency budget of the present PTM-enabled ISAC benchtop prototype.**

| **Component** | **Symbol** | **Representative latency** | **Notes** |
| --- | --- | --- | --- |
| PIN-diode intrinsic switching | $T_{\mathrm{PIN}}$ | **20 ns** | Intrinsic device-level switching |
| FPGA control update / bias refresh | $T_{\mathrm{FPGA}}$ | **7.6 µs** | Includes code update and bias refresh |
| VNA spectral acquisition | $T_{\mathrm{acq}}$ | **5 ms** | Dominant overhead in benchtop setup |
| Data transfer to host PC | $T_{\mathrm{transfer}}$ | **3 ms** | Depends on instrument interface |
| CNN single-sample inference | $T_{\mathrm{CNN}}$ | **0.19 ms** | One online feature input, not 111,000 entries |
| Communication-state triggering/link activation | $T_{\mathrm{comm}}$ | **0.17 ms** | Includes handoff to communication stage |
| **Total cycle** | $T_{\mathrm{total}}$ | **8.37 ms** | Sensing → recognition → switching → communication |

**Supplementary Note 15: Out-of-distribution robustness and deployment considerations**

The CNN-based positioning model presented in the main text is developed and validated in a controlled indoor environment. Therefore, its behavior under out-of-distribution (OOD) shifts, such as layout changes or substantially different target characteristics, deserves additional discussion. At the same time, the baseline dataset already contains several sources of variability: six volunteers with diverse body types participated in the experiments, posture variations were intentionally introduced during data collection, the sensing process involved six coding states, and the overall dataset comprised 111,000 entries. Moreover, the supplementary 222-class experiment further showed that the learned feature space remains highly discriminative across different subjects, coding states, positions, and unmanned conditions, with an overall accuracy of 98.57%. These results indicate that the CNN is learning structured topological spectral signatures rather than memorizing a single rigid sensing condition.

To further assess deployment-oriented robustness, we summarize four representative generalization settings: (1) cross-subject generalization, evaluated by leave-one-subject-out (LOSO) testing across the six volunteers; (2) furniture-shift OOD, representing moderate room-layout perturbation; (3) static-object OOD, representing the introduction of additional stationary scatterers into the monitored sub-regions; and (4) morphology-shift stress test, representing target characteristics that deviate from the main body-shape distribution seen during baseline training. The representative results are summarized in Table S3, Table S4 and Fig. S26.

The results show that the model maintains high localization accuracy under all tested conditions, while exhibiting a gradual performance decrease as the distribution shift becomes stronger. In particular, cross-subject testing remains highly accurate, indicating meaningful robustness to human-body variations within the current dataset. Layout changes and added static objects lead to moderate degradation, suggesting that the learned spectral features preserve useful position-dependent information under realistic scene perturbations, although they are not completely environment-invariant. The morphology-shift stress test shows a further but still controlled decrease, consistent with the expectation that larger deviations from the original training distribution require stronger adaptation.

These observations suggest that the full 111,000-entry dataset should be viewed as a research-grade benchmark for model development rather than a required per-site retraining burden for every deployment. In practical scenarios, a more realistic strategy would be to use the pretrained model as initialization and then perform site-specific calibration or fine-tuning with a smaller dataset, especially when the new installation differs only moderately from the original environment. For substantially changed room geometries or target distributions, additional adaptation data would likely be beneficial.

**Table S3 | Representative out-of-distribution robustness summary.**

| **Evaluation setting** | **Accuracy (%)** |
| --- | --- |
| Baseline single-volunteer test | 99.54 |
| Cross-subject LOSO (mean ± std) | 97.23 ± 0.44 |
| Furniture-shift OOD | 96.08 ± 0.72 |
| Static-object OOD | 95.60 ± 0.68 |
| Morphology-shift stress test | 95.17 ± 0.63 |
| Supplementary 222-class classification | 98.57 |

**Table S4 | Representative position-wise localization accuracy under OOD-type shifts (%).**

| **Position** | **Furniture shift** | **Static objects** | **Morphology shift** |
| --- | --- | --- | --- |
| Position 1 | 97.1 | 96.8 | 96.0 |
| Position 2 | 96.5 | 96.0 | 95.6 |
| Position 3 | 95.8 | 95.2 | 94.9 |
| Position 4 | 95.1 | 94.8 | 94.4 |
| Position 5 | 96.3 | 95.7 | 95.3 |
| Position 6 | 95.7 | 95.1 | 94.8 |

**Fig. S26 | Out-of-distribution robustness of the PTM-enabled positioning framework. a** Overall localization accuracy under baseline, cross-subject leave-one-subject-out (LOSO), furniture-shift OOD, static-object OOD, and morphology-shift stress-test conditions. **b** Position-wise localization accuracy under representative OOD-type shifts, showing that the proposed framework remains robust while exhibiting moderate performance degradation under stronger distribution changes.

**Supplementary Note 16: System-level power efficiency and hardware-complexity considerations**

The main architectural advantage of the proposed PTM-enabled ISAC platform is the reuse of a single programmable aperture for both sensing and communication, thereby avoiding the duplication of physically separate sensing and communication modules. However, because the present proof-of-concept setup is implemented using benchtop instruments such as a VNA, a PC, an FPGA controller, and a USRP, a direct whole-system PAE comparison is not the most suitable metric. In particular, the VNA is used here as a laboratory sensing/data-acquisition instrument and should not be interpreted as the final sensing front-end in a future deployment-oriented implementation.

For this reason, we evaluate the issue from two complementary perspectives. First, we provide a representative deployment-oriented total active power comparison between the proposed PTM-based unified-aperture architecture and a conventional ISAC architecture employing separate sensing and communication apertures/modules. The comparison is intended to highlight the architectural impact of aperture reuse and reduced hardware duplication, rather than to report a strict measured power budget of the benchtop prototype. The total active power is written as

$$\begin{aligned} P_{\mathrm{sys}}=\sum_{i} P_{i},\#\left( S12 \right) \end{aligned}$$

where $P_{i}$ denotes the power contribution of each major hardware block. Table S5 summarizes a representative module-level comparison. The PTM-based approach uses one shared programmable aperture and one unified control architecture, whereas the conventional architecture uses separate sensing and communication apertures and correspondingly larger front-end/control overhead. As a representative engineering estimate, the PTM-based architecture yields a total active power of 8.50 W, compared with 11.75 W for the separate-aperture benchmark, corresponding to an approximate 27.7% reduction.

Second, we assess the impact of the present 1-bit phase modulation on radiative efficiency. For an ideal B-bit quantized phase aperture, the normalized phase-quantization efficiency can be approximated by

$$\begin{aligned} \eta_{q}(B)=\left[ \frac{\sin(\pi/2^{B})}{\pi/2^{B}} \right]^{2}.\#\left( S13 \right) \end{aligned}$$

For the current PTM, the measured phase contrast between Units 0 and 1 is about $\Delta\phi\approx140\circ$ at 6.2 GHz rather than an ideal $180\circ$. We therefore introduce an additional binary phase-contrast factor

$$\begin{aligned} \eta_{\Delta}=\sin^{2}\left( \frac{\Delta\phi}{2} \right),\#\left( S14 \right) \end{aligned}$$

so that the effective normalized coherent beamforming efficiency of the current 1-bit PTM can be estimated as

$$\begin{aligned} \eta_{\mathrm{PTM}}\approx\eta_{q}(1)\eta_{\Delta}\approx0.405\times\sin^{2}({70}^{\circ})\approx0.358.\#\left( S15 \right) \end{aligned}$$

This corresponds to an approximate SNR penalty of

$$\begin{aligned} \Delta SNR\approx10\log_{10}(\eta_{\mathrm{PTM}})\approx-4.46\mathrm{dB}\#\left( S16 \right) \end{aligned}$$

relative to an ideal continuous-phase aperture. Table S6 summarizes the comparison with higher-bit programmable metasurfaces. These results indicate that the current 1-bit PTM sacrifices some beamforming efficiency and SNR margin in exchange for reduced biasing complexity, lower control overhead, and simpler hardware implementation. This trade-off is appropriate for the present proof-of-concept, while higher-resolution phase control is a promising direction for future optimization.

**Fig. S27 | Power-complexity and phase-efficiency comparison. a** Representative module-level active power comparison between the PTM-based unified-aperture architecture and a conventional separate sensing/communication architecture. **b** Analytical comparison of normalized coherent beamforming efficiency for the present 1-bit PTM and higher-resolution phase-control schemes.

**Table S5 | Representative deployment-oriented total active power comparison.**

| **Category** | **PTM-based unified-aperture ISAC (W)** | **Conventional separate sensing + communication architecture (W)** |
| --- | --- | --- |
| Programmable aperture / bias network | 0.65 | 1.40 |
| Sensing RF front-end | 1.85 | 2.80 |
| Communication RF/IF chain | 3.60 | 4.20 |
| Control / synchronization | 1.35 | 1.90 |
| Baseband / processing | 1.05 | 1.45 |
| **Total active power** | **8.50** | **11.75** |

**Table S6 | Analytical efficiency/SNR comparison versus phase resolution.**

| **Configuration** | **Effective phase contrast** | **Normalized beamforming**  **efficiency η** | **Approx. SNR penalty vs. continuous phase** |
| --- | --- | --- | --- |
| Present PTM (1-bit, actual) | 140∘ | **0.358** | **-4.46 dB** |
| Ideal 1-bit | 180∘ | **0.405** | **-3.92 dB** |
| Ideal 2-bit | ideal quantized | **0.811** | **-0.91 dB** |
| Ideal 3-bit | ideal quantized | **0.950** | **-0.22 dB** |
| Continuous phase | continuous | **1.000** | **0 dB** |

**Supplementary Note 17: Transient interference mitigation and mode isolation during state switching**

The proposed PTM-enabled ISAC platform operates in a sequential closed-loop manner, in which the sensing state and the communication state are separated in time. Therefore, the key issue during state transition is not long-term simultaneous interference, but whether the short reconfiguration window introduces residual transient leakage that could affect the subsequent communication link. To clarify this point, we introduce a representative transition-isolation analysis from two perspectives. First, we define the steady-state cross-mode isolation as the suppression of undesired communication-channel response in the non-trivial sensing state and the suppression of undesired sensing-path response in the trivial communication state. Second, we define the residual transient leakage during the reconfiguration interval before the communication trigger is enabled.

Figure S28a illustrates the gated timing sequence of the present workflow. After sensing acquisition and classification, the FPGA updates the PTM state. A short guard interval is then inserted before the USRP payload transmission starts. Figure S28b shows representative normalized transient power traces of the decaying surface-wave sensing mode, the rising spatial-wave communication mode, and the residual mixed-mode leakage. The results indicate that the mixed-mode transient is confined to a short transition window and decays rapidly before the communication trigger point.

The quantitative results are summarized in Table S7. The representative steady-state cross-mode isolation is about 23–26 dB, while the residual transient leakage is reduced to below −30 dB before the communication payload is enabled. These results support the statement in the main text that the trivial–nontrivial transition effectively mitigates mutual interference, more specifically through time separation, trigger gating, and finite transition settling rather than through simultaneous dual-mode coexistence.

**Table S7 | Representative transition-isolation metrics of the PTM-enabled ISAC workflow.**

| **Metric** | **Representative value** | **Physical meaning** |
| --- | --- | --- |
| Sensing-state $\to$ communication-channel leakage | **−23.8 dB** | Undesired radiative response at the designated communication receiver when PTM stays in the non-trivial sensing state |
| Communication-state $\to$ sensing-path leakage | **−25.6 dB** | Undesired signal coupled to the straight sensing path when PTM stays in the trivial communication state |
| Peak transient mixed-mode component during switching | **−17.2 dB** | Short-lived overlap during the reconfiguration window |
| Residual transient leakage at (t=0.8$\mu s$) | **−29.6 dB** | Leakage after electrical settling begins to complete |
| Residual transient leakage at USRP trigger instant ((t=1.0$\mu s$) | **−33.2 dB** | Residual leakage before payload transmission |
| Recommended guard interval before payload enabling | **1.0** $\boldsymbol{\mu}s$ | Representative control margin for transition isolation |

**Fig. S28 | Representative transition-isolation analysis during PTM state switching. a** Gated control timeline of sensing, FPGA reconfiguration, guard interval, and communication triggering. **b** Representative transient power evolution of the decaying sensing mode, rising communication mode, and residual mixed-mode leakage during the switching window. **c** Quantitative summary of steady-state and transition isolation metrics.

**Supplementary Note 18 Benchmarking against representative literature and discussion of mode purity / blind-spot considerations**

A direct numerical benchmark of the proposed PTM-based sensing platform against existing literature^21-28^ should be interpreted with care, because different communities often report different performance metrics. In the present work, the main sensing result is a six-region classification accuracy of 99.54% for near-field human localization, whereas many representative UWB positioning studies report continuous-coordinate localization error in centimeters, and most topological sensing studies report scalar sensing metrics such as refractive-index sensitivity, temperature sensitivity, or quality factor. Therefore, the purpose of the present comparison is not to claim a strict one-to-one superiority under a unified benchmark, but rather to situate the proposed PTM approach within the broader research landscape.

Recent reviews of topological photonic sensors show that the field has been dominated by applications such as refractive-index sensing, biosensing, gas sensing, and thermal sensing, rather than near-field human localization. Representative topological sensor studies report, for example, dual-parameter sensitivities of 653 nm/RIU and 235 pm/°C, or thermal sensing performance of 1.62 nm/°C with high quality factor and SNR. In this context, the present work differs fundamentally in task definition: it uses topologically protected surface-wave routing to extract human-position-dependent electromagnetic signatures for intelligent localization.

Representative UWB-based human-sensing and localization literature provides useful context from the application side. Depending on the task and setup, UWB-related studies have reported values such as 85% posture-classification accuracy, ~95% movement-classification accuracy, 96.1% real-time pose-detection accuracy, and localization errors of 20 cm, 12.23 cm, 6.69 cm (NLOS), and 2.49 cm (LOS). These results indicate that UWB is a strong benchmark for near-field/indoor human sensing, but they also highlight that the reported metric often differs from the six-region classification accuracy used in the present work.

It is also important to clarify the possible “blind-spot” issue in the PTM architecture. In the present system, the sensing mode and the communication mode are realized by sequential state switching, not by simultaneous mixed-state operation. During sensing, the topological domain-wall network is formed by the nontrivial mirror-related unit pair (Units 2 and 3), whereas the trivial unit pair (Units 0 and 1) is used only after reconfiguration to the communication state. Therefore, there are no fixed sensing regions where topological protection is intrinsically weakened due to the proximity of trivial communication units. The one-to-two routing field distributions and the high classification results suggest that the current six-region sensing layout does not exhibit obvious blind spots. Any remaining spatial nonuniformity is more reasonably attributed to terminal geometry, local field coverage, and target-to-terminal distance than to a breakdown of topological protection.

Overall, this comparison suggests that the proposed PTM platform is not merely another high-accuracy classifier, but a distinct sensing architecture that introduces topologically protected surface-wave routing into near-field ISAC. Its significance lies in combining competitive sensing performance with a unified programmable aperture that can subsequently transition to directional communication without additional hardware.

**Table S8 | Representative comparison with topological sensing literature.**

| **Work** | **Modality / platform** | **Task type** | **Reported metric** | **Representative result** |
| --- | --- | --- | --- | --- |
| Sheng et al. (2025) | Valley-Hall photonic crystal sensor | Dual-parameter RI/temperature sensing | Sensitivity | 653 nm/RIU; 235 pm/°C |
| Entezar (2025) | 1D topological photonic crystal | Thermal sensing | Sensitivity / Q / SNR | 1.62 nm/°C; (Q$\sim$ 19899); SNR ($\sim$ 34.5) |
| This work | PTM-based topological metasurface | Near-field human localization | Six-region classification accuracy | **99.54%** |

**Table S9 | Representative comparison with UWB-based human-sensing / localization literature.**

| **Work** | **Sensing modality** | **Scenario** | **Reported metric** | **Representative result** |
| --- | --- | --- | --- | --- |
| Baird et al. (2018) | UWB radar | Indoor posture classification | Overall accuracy | 85% |
| Lee et al. / Noh et al. (2024) | UWB sensors | Human movement recognition | Average classification accuracy | ~95% |
| Choi & Bhattacharya (2024 / GLOBECOM 2023) | UWB tagless gate | Real-time smartphone pose detection | Pose accuracy | 96.1% |
| Ridolfi et al. (2018) | UWB indoor positioning | Sport postures / dynamic users | Average localization error | 20 cm |
| Tu et al. (2024) | NN-enhanced UWB | Indoor multi-obstacle localization | Average positioning error | 12.23 cm |
| Lou et al. (2022) | UWB + digital twin + NN mitigation | LOS / NLOS indoor positioning | Average localization error | 2.49 cm (LOS); 6.69 cm (NLOS) |
| This work | PTM-based topological metasurface | Six-region near-field human localization | Classification accuracy | **99.54%** |

**Fig. S29| Benchmark overview against representative UWB-based human-sensing literature. a** Comparison of representative classification-style metrics reported for near-field / human-sensing tasks. **b** Comparison of representative UWB indoor localization errors reported in the literature. Lower values indicate better coordinate localization precision.

**Supplementary References**

1.	Wang, Z., Chong, Y., Joannopoulos, J.D. & Soljačić, M. Observation of unidirectional backscattering-immune topological electromagnetic states. *Nature* **461**, 772-775 (2009).

2.	Wang, Z., Chong, Y.D., Joannopoulos, J.D. & Soljačić, M. Reflection-Free One-Way Edge Modes in a Gyromagnetic Photonic Crystal. *Physical Review Letters* **100**, 013905 (2008).

3.	Raghu, S. & Haldane, F.D.M. Analogs of quantum-Hall-effect edge states in photonic crystals. *Physical Review A* **78**, 033834 (2008).

4.	Wu, L.-H. & Hu, X. Scheme for Achieving a Topological Photonic Crystal by Using Dielectric Material. *Physical Review Letters* **114**, 223901 (2015).

5.	Yang, Y.*, et al.* Visualization of a Unidirectional Electromagnetic Waveguide Using Topological Photonic Crystals Made of Dielectric Materials. *Physical Review Letters* **120**, 217401 (2018).

6.	Hafezi, M., Demler, E.A., Lukin, M.D. & Taylor, J.M. Robust optical delay lines with topological protection. *Nature Physics* **7**, 907-912 (2011).

7.	Dong, J.-W., Chen, X.-D., Zhu, H., Wang, Y. & Zhang, X. Valley photonic crystals for control of spin and topology. *Nature Materials* **16**, 298-302 (2017).

8.	Xiao, D., Chang, M.-C. & Niu, Q. Berry phase effects on electronic properties. *Reviews of Modern Physics* **82**, 1959-2007 (2010).

9.	Xiao, Q.*, et al.* Programmable topological metasurface to modulate spatial and surface waves in real time. *Nanophotonics* **13**, 2141-2149 (2024).

10.	Zhao, R.*, et al.* First-principle calculation of Chern number in gyrotropic photonic crystals. *Opt. Express* **28**, 4638-4649 (2020).

11.	Li, J., Wu, Q. & Weng, H. High-Chern-number Quantum anomalous Hall insulators in mixing-stacked MnBi2Te4 thin films. *npj Quantum Materials* **10**, 53 (2025).

12.	Zeng, Y. Tunable fractional Chern insulators. *Nature Materials* (2025).

13.	Park, H.*, et al.* Ferromagnetism and topology of the higher flat band in a fractional Chern insulator. *Nature Physics* **21**, 549-555 (2025).

14.	Nuckolls, K.P.*, et al.* Strongly correlated Chern insulators in magic-angle twisted bilayer graphene. *Nature* **588**, 610-615 (2020).

15.	Redekop, E.*, et al.* Direct magnetic imaging of fractional Chern insulators in twisted MoTe2. *Nature* **635**, 584-589 (2024).

16.	Bleu, O., Malpuech, G. & Solnyshkov, D.D. Robust quantum valley Hall effect for vortices in an interacting bosonic quantum fluid. *Nature Communications* **9**, 3991 (2018).

17.	Zhang, Q.*, et al.* Gigahertz topological valley Hall effect in nanoelectromechanical phononic crystals. *Nature Electronics* **5**, 157-163 (2022).

18.	Tian, H.W.*, et al.* Solar-powered light-modulated microwave programmable metasurface for sustainable wireless communications. *Nature Communications* **16**, 2524 (2025).

19.	Wu, H.*, et al.* A programmable metasurface antenna that approaches the wireless information mapping limit. *Nature Electronics* **8**, 179-191 (2025).

20.	Li, F.*, et al.* Flexible intelligent microwave metasurface with shape-guided adaptive programming. *Nature Communications* **16**, 3161 (2025).

21.	Sheng, G.*, et al.* Topologically-protected dual-parameter Valley Hall photonic crystal sensor. *Opt. Express* **33**, 25558-25569 (2025).

22.	Roshan Entezar, S. One-dimensional topological photonic crystal-based thermal sensor. *Appl. Opt.* **64**, 6672-6680 (2025).

23.	Baird, Z., Rajan, S. & Bolic, M. Classification of Human Posture from Radar Returns Using Ultra-Wideband Radar. in *2018 40th Annual International Conference of the IEEE Engineering in Medicine and Biology Society (EMBC)* 3268-3271 (2018).

24.	Noh, M., Ahn, H. & Lee, S.C. Real-Time Human Movement Recognition Using Ultra-Wideband Sensors. *Electronics* **13**, 1300 (2024).

25.	Choi, J. & Bhattacharya, S. Deep Learning-Based Real-Time Smartphone Pose Detection for Ultra-Wideband Tagless Gate. in *GLOBECOM 2023 - 2023 IEEE Global Communications Conference* 4595-4600 (2023).

26.	Ridolfi, M.*, et al.* Experimental Evaluation of UWB Indoor Positioning for Sport Postures. *Sensors* **18**, 168 (2018).

27.	Lou, P., Zhao, Q., Zhang, X., Li, D. & Hu, J. Indoor Positioning System with UWB Based on a Digital Twin. *Sensors* **22**, 5936 (2022).

28.	Tu, C., Zhang, J., Quan, Z. & Ding, Y. UWB indoor localization method based on neural network multi-classification for NLOS distance correction. *Sens. Actuators, A* **379**, 115904 (2024).
